# Localization and abundance analysis of human lncRNAs at single-cell and single-molecule resolution

**DOI:** 10.1186/s13059-015-0586-4

**Published:** 2015-01-29

**Authors:** Moran N Cabili, Margaret C Dunagin, Patrick D McClanahan, Andrew Biaesch, Olivia Padovan-Merhar, Aviv Regev, John L Rinn, Arjun Raj

**Affiliations:** Broad Institute of MIT and Harvard, 7 Cambridge Center, Cambridge, MA 02142 USA; Department of Systems Biology, Harvard Medical School, Boston, MA 02115 USA; Department of Stem Cell and Regenerative Biology, Harvard University, Cambridge, MA 02138 USA; School of Engineering and Applied Science, University of Pennsylvania, Philadelphia, PA 19104 USA; Howard Hughes Medical Institute, Department of Biology, Massachusetts Institute of Technology, Cambridge, MA 02140 USA

## Abstract

**Background:**

Long non-coding RNAs (lncRNAs) have been implicated in diverse biological processes. In contrast to extensive genomic annotation of lncRNA transcripts, far fewer have been characterized for subcellular localization and cell-to-cell variability. Addressing this requires systematic, direct visualization of lncRNAs in single cells at single-molecule resolution.

**Results:**

We use single-molecule RNA-FISH to systematically quantify and categorize the subcellular localization patterns of a representative set of 61 lncRNAs in three different cell types. Our survey yields high-resolution quantification and stringent validation of the number and spatial positions of these lncRNA, with an mRNA set for comparison. Using this highly quantitative image-based dataset, we observe a variety of subcellular localization patterns, ranging from bright sub-nuclear foci to almost exclusively cytoplasmic localization. We also find that the low abundance of lncRNAs observed from cell population measurements cannot be explained by high expression in a small subset of ‘jackpot’ cells. Additionally, nuclear lncRNA foci dissolve during mitosis and become widely dispersed, suggesting these lncRNAs are not mitotic bookmarking factors. Moreover, we see that divergently transcribed lncRNAs do not always correlate with their cognate mRNA, nor do they have a characteristic localization pattern.

**Conclusions:**

Our systematic, high-resolution survey of lncRNA localization reveals aspects of lncRNAs that are similar to mRNAs, such as cell-to-cell variability, but also several distinct properties. These characteristics may correspond to particular functional roles. Our study also provides a quantitative description of lncRNAs at the single-cell level and a universally applicable framework for future study and validation of lncRNAs.

**Electronic supplementary material:**

The online version of this article (doi:10.1186/s13059-015-0586-4) contains supplementary material, which is available to authorized users.

## Background

Deep-sequencing based studies have revealed thousands of long non-coding RNAs (lncRNAs) expressed from mammalian genomes. While a number of studies have implicated functional roles lncRNAs [1-3] the vast majority remain uncharacterized [4,5]. Even very basic properties such as subcellular localization or absolute abundance in single cells remain unknown.

Knowledge of lncRNA subcellular localization patterns can provide fundamental insights into their biology and fosters hypotheses for potential molecular roles. Unlike mRNAs, which produce proteins, lncRNA themselves must localize to their particular site of action, making their location within the cell important. For instance, exclusively nuclear localization would argue against putative lncRNAs encoding short peptide sequences, because translation occurs in the cytoplasm. Further, localization to particular areas within the nucleus may suggest different functionalities - for instance, finding a lncRNA primarily in the nucleus near its site of transcription may suggest that it regulates transcription of a proximal gene (that is, regulation in *cis* or regulation of proximal loci in three dimensions) [6-8]. Sequencing studies cannot discriminate these possibilities, and so there is as yet no systematic categorization of lncRNA localization patterns.

The absolute abundance of lncRNAs in single cells is also subject to debate, but has critical implications for the stoichiometry of molecular mechanisms. On the whole, the expression of most lncRNAs tends to be lower than that of mRNA [9], and so their total abundance is likely far lower than that of proteins, which greatly restricts the number of sites at which a lncRNA may be active. One hypothesis [10] is that despite a low average abundance of lncRNAs, small numbers of cells in the population may express high numbers of lncRNA, thereby allowing for an increased number of sites of action in those cells. This hypothesis, however, has not yet been subjected to rigorous examination.

RNA fluorescence *in situ* hybridization (RNA FISH) [11,12] is an approach that can address these questions and suggest potential mechanisms for lncRNA activity. Indeed, direct observation of lncRNA localization by RNA FISH led to many of the early hypotheses about lncRNA function that now serve as paradigms in the field. An early example is the lncRNA XIST [13,14], a key regulator of X inactivation [15], in which RNA FISH demonstrated that XIST accumulates on the inactive X-chromosome [6,7]. Other more recent examples include MALAT1, NEAT1, and MIAT (Gomafu) which are localized to nuclear bodies [16-20] and the lncRNA GAS5 which shuttles between the nucleus and cytoplasm [21]. One notable early study surveyed lncRNA expression in brain at tissue level resolution using these *in situ* hybridization techniques [22]. These examples are, however, among the mostly highly abundant RNAs in the cell, whereas the vast majority of lncRNAs are considerably less abundant [9], precluding the use of conventional RNA FISH techniques that have relatively low sensitivity.

More recently, researchers have developed and applied single molecule RNA FISH techniques based on hybridization of multiple short, fluorescently labeled, oligonucleotide probes [23,24] to estimate the absolute level and subcellular localization of even low abundance lncRNAs [8,25-31]. Single-cell correlations between a lncRNA and its putative mRNA target (simultaneously monitored with two differently colored fluorescent dyes) can suggest potential regulatory interactions [27,32]. For instance, combining correlation analysis with subcellular localization revealed that lncHOXA1 represses the neighboring Hoxa1 gene in *cis* in a subpopulation of cells, a finding made possible by directly visualizing lncRNA activity at the site of transcription [8].

Yet, no study has systematically applied single molecule RNA FISH to explore lncRNA localization and abundance from cDNA and RNA-seq catalogs, such as those in [9,33-38]. Furthermore, no study has systematically tackled the unique technical challenges posed by performing single molecule RNA FISH on lncRNAs, which are shorter, lower abundance and more likely to contain repeats than mRNA [9,39].

Here, we used single molecule RNA FISH in single cells to characterize the sub-cellular localization patterns and abundance of 61 lncRNAs across three human cell types. We focused on the subclass of intergenic lncRNAs (lincRNAs) [40] from our well-annotated Human lincRNA Catalog [9], and systematically selected a subset spanning a wide range of tissue specificity and expression levels while encompassing both syntenically orthologous lincRNAs [9,37] and divergently transcribed lincRNAs [9,35,41-43].

Our first observation was that lncRNA FISH is prone to artifacts (likely owing to low abundance and repetitive nature of lncRNAs), and so we established a pipeline for rigorous validation of single molecule RNA FISH probe sets. Once established, this approach allowed us to address several fundamental questions about lncRNA biology. First, lncRNAs exhibited a wide range of subcellular localization patterns, including distinct categories of nuclear localization, with most lncRNAs showing stronger nuclear localization than most mRNAs. In most cases, these localization patterns were consistent across the three different cell types tested. Second, we found that the low abundance of lncRNAs in bulk population measurements is not due to a small subpopulation of cells expressing lncRNAs at high-levels, and overall lncRNA are no different than mRNA in their levels of cell-to-cell heterogeneity. Third, we found that in mitotic cells, lncRNAs do not associate with chromatin, showing that (at least for the examined cases) retention at specific regulatory regions through mitosis is likely not a mechanism of mitotic inheritance. Finally, simultaneous analysis of matching pairs of divergently transcribed lncRNAs and mRNAs showed that these pairs are not always co-regulated and that the localization patterns of divergently transcribed lncRNA do not differ from those of other lncRNAs. Taken together, these finding describe the fundamental properties of lncRNA’s cell-to-cell expression variability and establish a canonical set of patterns of lncRNA localization.

## Results

### A single molecule, single cell RNA FISH survey of lncRNAs in three human cell types

**Figure 1:**
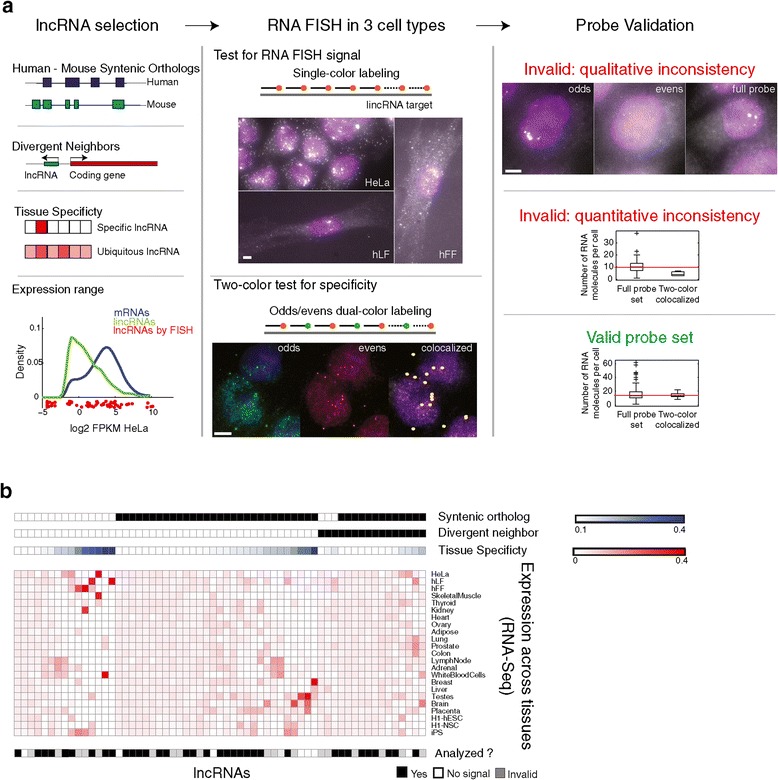
**An RNA-FISH survey of lncRNAs. (a)** Study workflow. **(b)** Key features of 61 lincRNAs for which probe sets were successfully designed and were imaged in the study. Shown are for each of 61 lincRNA (column) the following features from top to bottom: whether it has a syntenic ortholog (black: has ortholog) or a divergently transcribed mRNA neighbor (black: has such neighbor), the extent of tissue specificity across 23 tissues (blue color intensity: maximal tissue specificity score as in [9] across the tissues presented in the figure; white to blue color bar), its expression level as measured by RNA-Seq (red intensity: the fractional density across the row of log_2_(FPKM) as estimated by Cufflinks; white to red color bar) in each of 23 tissues (heatmap rows; Additional file 2), and the extent of analysis performed (black: lncRNAs with valid probe set that were included in the final analysis; white: lncRNAs showing no signal; gray: lncRNAs with an invalid probe based on the two-color co-localization assay).

To characterize the abundance and localization patterns of lncRNAs in the three different cell types, we studied 61 lncRNAs systematically selected to span a range of parameters (Figure 1a) using single molecule RNA FISH. Specifically, we manually curated a candidate set of 61 lncRNA for screening (Figure 1; Additional files 1 and 2) such that: (1) the lncRNAs in our set are significantly expressed in at least one of human foreskin fibroblasts (hFFs), human lung fibroblasts (hLFs), or HeLa cells, the target cell lines for our study; (2) the lncRNAs span a wide range of expression levels and tissue specificity (Additional file 1: Figure S1; Additional file 2); (3) the set includes a subset of 43 lncRNAs that have an expressed syntenic ortholog in mouse; and (4) the set includes a subset of 16 lincRNAs that are transcribed divergently to a neighboring mRNA (within 10 KB). These criteria and subsets are not mutually exclusive (Figure 1b). Finally, we included 16 previously studied lncRNAs as a point of reference. We also included two different groups of mRNA controls (Additional file 3; 34 in total): (1) nine mRNAs transcribed divergently to those ‘divergent lncRNAs’ in this study the cyclin CCNA2 as a marker of cell cycle; and (2) 24 mRNAs that span a wide range of expression levels in hFF (Padovan-Merhar and Raj, personal communication).

To visualize single lncRNA molecules directly inside of cells, we used an established protocol for single molecule RNA FISH [24], where we design 10 to 48 complementary DNA oligonucleotides, each 20 bases long and labeled with a single fluorophore at its 3′ end (Figure 1a). When these probes hybridize to a single RNA molecule, the concentration of so many fluorophores at a single location renders the RNA molecule detectable by fluorescence microscopy. When applied to mRNAs, this method has typically been proven highly specific, as signal is only detectable when a large fraction of the probe set hybridizes to the target [24], and is highly accurate as gauged by quantitative polymerase chain reaction (qPCR) [44-48]. We successfully designed probe sets for 61 lncRNAs in hFFs, hLFs, and HeLa cells (Methods; Additional file 3), 53 of which yielded a detectable signal in at least one cell type. In all of the hybridizations we performed, we co-stained for CCNA2 mRNA, a cyclin whose transcripts are present only in S/G2/M, thus providing us with cell cycle information for the cells we imaged.

During the course of our investigations, we noticed that performing RNA FISH on lncRNAs presented a major challenge due to off-target binding of oligonucleotides. Even a single oligonucleotide binding to a highly abundant off-target RNA can lead to spurious signals, problems exacerbated by lncRNAs’ higher repeat content [39] (leading to more potential off-targets) and typically lower abundance than mRNAs [9] (making off-target binding more noticeable). For example, we noticed images of a particular lncRNA with similar localization patterns to MALAT1; however, removal of just one oligonucleotide from the probe pool with homology to MALAT1 resulted in complete loss of the dominant signal (Additional file 1: Figure S2a).

To control for these ‘rogue’ oligonucleotides with off-target signal, we used a two-color co-localization approach [23,24] in which we analyzed each lncRNA after partitioning its probe set into two subsets (‘even’ and ‘odd’ oligonucleotides), each labeled with a differently colored fluorophore (Figure 1a; Additional file 1: Figure S2b-d; Methods). If the oligonucleotides in the probe set were binding specifically, the signals from these two subsets should largely co-localize (for example, Figure 1a middle; Additional file 1: Figure S2b), with the number of co-localized spots roughly equaling those obtained from the full probe set (‘quantitative consistency’; Figure 1a right; Additional file 1: Figure S2d). If a single oligonucleotide hybridizes to a highly abundant off target, we would see the signal only in either the odd or even channel (see for example Figure 1a right or Additional file 1: Figure S2c for an ‘invalid’ probe set targeting). Note that for mRNA, the presence of nuclear bright foci of off-target signal is less of a concern than for lncRNA because they seldom display such bright foci without also exhibiting very large numbers of cytoplasmic RNA, whereas for lncRNA, we have found several examples for which the legitimate signal can take on this pattern (for example, Xist, Kcnq1ot1 [6,28]). We also observed cases in which the number of spots in the full probe set differed dramatically from the number of co-localized spots, potentially indicating some other non-specific background (‘quantitative inconsistency’, Figure 1a right; Additional file 1: Figure S2c).

Using the ‘two-color co-localization’ validation, we eliminated 19 probe sets from further analysis, as they had major qualitative or quantitative differences in the two color co-localization assay, underscoring the importance of testing for off-target effects for lncRNA FISH (Figure 1a; Additional file 1: Figure S2d-e and Figure S21; Additional file 4). Another eight probe sets had no discernible signal in any of the three examined cell types. We were unable to attribute the cases of no detectable signal or co-localization inconsistencies to low number of oligonucleotides and observed a very slight bias toward lower abundance lncRNAs (Kruskal-Wallis one way analysis of variance *P* <8.4X10^-3^; Additional file 1: Figure S3). Importantly, our validation approach was required in each cell type investigated, as some probes were valid in one cell type but not in another (Additional file 1: Figure S4). Upon further checking for quantitative consistency (Methods; Additional file 1: Figure S1a, Figure S2e, Figure S21; Additional file 4), we were left with 70 lncRNA-cell type pairs with valid signal, corresponding to 34 unique lncRNAs (Additional file 4; Additional file 1: Figure S22). Altogether, we acquired over 2,000 images overall in three to five separate fluorescence channels, with two to three biological replicates per gene-cell pair (the final analysis included 80, 24, and 28 cells per gene on average, for HeLa cells, hLFs, and hFFs, respectively).

### lncRNAs exhibit a diversity of localization patterns composed of a few basic characteristics

**Figure 2:**
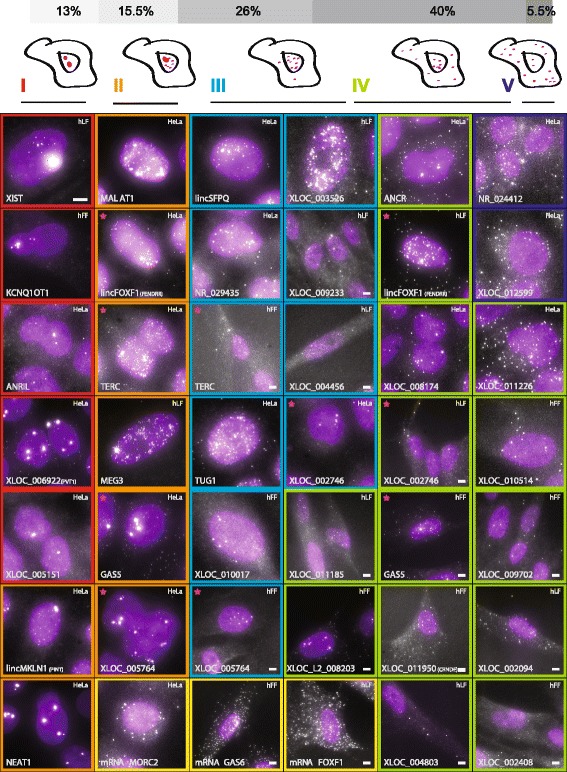
**LncRNAs exhibit a variety of cellular localization patterns.** Florescence micrographs of representative expressing cells for each of 34 lncRNAs with a validated probe set. LncRNA-cell pairs are classified to cellular localization types I to V as described in the Methods (marked by their border color). Magenta stars mark five lncRNAs that are presented in two different cell types and two different classes (see same row for comparison). Scale bar, 5 μm; when a scale bar is not specified, reference the scale bar within the top left image. Top panel: fraction of each classification for each type across the full set of 70 valid lncRNA-cell pairs imaged.

We examined the cytoplasmic and nuclear localization of these 34 lncRNAs in the three cell types (70 lncRNA-cell type pairs) and observed a wide range of localization patterns (Figure 2; Additional file 1: Figure S5). These patterns consisted of combinations of a few basic features, including bright nuclear foci with multiple RNA in them, monodisperse single RNAs in the nucleoplasm, and monodisperse single RNAs in the cytoplasm. The bright nuclear foci also took a number of different forms: most consisted of a few tight puncta, but some exhibited a spatial delocalization, such as XIST, or many bright accumulations, such as MALAT1. We did not observe bright accumulations of lncRNA in the cytoplasm. These features did not manifest independently - for instance, the presence of nuclear foci was typically associated with more nuclear than cytoplasmic spots. Thus, we classified the lncRNA into the following types: (Methods; Additional file 5): (I) one or two large foci in the nucleus (nine pairs); (II) large nuclear foci and single molecules scattered through the nucleus (11 pairs); (III) predominantly nuclear, without foci (18 pairs); (IV) cytoplasmic and nuclear (28 pairs); and (V) predominantly cytoplasmic (four pairs). Validating our approach, 11 of the 12 lncRNA previously imaged by RNA FISH [6,19,21,25,49-56] showed patterns that were consistent with previous reports (Additional file 3). These included the large nuclear foci previously observed for XIST and Kcnq1ot1 [6,7,51], localization of GAS5 to both the nucleus and cytoplasm [21] and the speckle- and para-speckle-like structures of MALAT1 and NEAT1, respectively [19,49].

The majority of lncRNAs (55% classified as class I to III; 38 lncRNA-cell type pairs) are predominantly in the nucleus (Additional file 1: Figure S3a and b; Methods; compared to 1/49 of mRNAs using the I to III classification criteria of more than 65% of molecules in the nucleus), with approximately 13% of lncRNA-cell type pairs mainly located in one or two large foci (type I). As noted, we also observed two distinct types of nuclear localization patterns: (1) localization to tight foci in the nucleus (for example, XLOC_006922, XLOC_005764); and (2) a more diffuse but spatially ‘speckled’ pattern (for example, MALAT1, MEG3, XLOC_003526). Interestingly, using simultaneous imaging of MALAT1, MEG3, and XLOC_003526 by labeling each target with different fluorescent dye in hLFs and hFFs, we find that the three lncRNA share a ‘speckle like’ localization pattern, and a significant fraction of MEG3 molecules co-localize with MALAT1 (statistically significant overlap in approximately 80% of cells examined; Additional file 1: Figure S6, Methods; Additional file 5).

**Figure 3:**
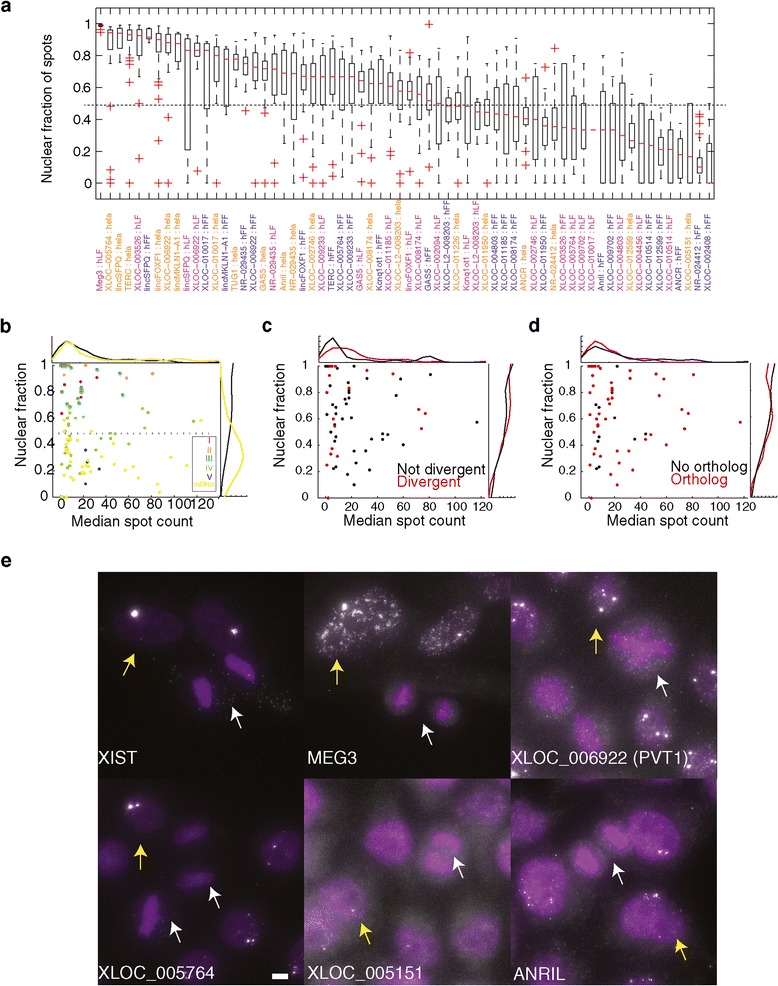
**Most lincRNAs are predominantly localized to the nucleus. (a)** Boxplots describing the distribution of the fraction of molecules localized to the nucleus (Y axis) for each validated lncRNA-cell pair (X axis, orange: HeLa, blue: hFF, purple: hLF). Red bar: medians. Whiskers are at 1.5* the inner quartile range. **(b)** Scatter plot of the relationship between expression level (X axis; median number of molecules per cell) and nuclear localization (Y axis, median fraction of nuclear spots across all expressing cells). Each data point is one gene-cell pair and is colored by its classification to the localization classes I to V (Methods) of Figure 2. mRNA sets 1 to 2 (yellow) serve as a reference. Histograms on top and right are the distribution of all lncRNAs- (black) and mRNA- (yellow) cell pairs. **(c)** Scatter and histograms as in (b) but for lncRNA with (red) or without (black) a divergently transcribed mRNA counterpart. **(d)** Scatter and histograms as (b) but for lncRNA with (red) or without (black) a syntenic ortholog. **(e)** Representative image of mitotic cells (marked with white arrows) lacking foci that are seen in interphase cells (marked with yellow arrows). Scale bar, 5 μm.

The bias toward nuclear localization was significant compared to localization of mRNAs (67% of lncRNAs vs. 10% of mRNAs have more than 50% of their RNA in the nucleus; Kolmogorov Smirnov (KS) *P* <13×10^-11^; Figure 3a and b). Within the lncRNA set, divergent lncRNAs presented a slightly higher bias toward nuclear localization (KS *P* <2.12×10^-2^; effect size = 0.35; Figure 3c) while syntenic orthologs did not present such bias over the lncRNA background distribution. The latter set did, however, exhibit a slight bias toward higher expression (KS *P* <3.25×10^-3^; Figure 3d).

In the vast majority (85%) of cases, the lncRNA localization pattern was consistent across the cell types where data were available. The notable exceptions were five lncRNAs (lincFOXF1, TERC, XLOC_005764, GAS5, XLOC_002746) that displayed distinct patterns in at least two cell types. These differences, however, appeared mostly to result from differences in overall abundance that likely leads to the appearance of additional bright foci in the nucleus (Figure 2, magenta stars, Additional file 1: Figure S7, S8, S9; Additional file 5). For example, we identified large lncRNA foci for TERC and XLOC_005764 in HeLa cells (type II), where they are more abundant (approximately 81 and 22 molecules per cell, respectively) than in hFFs (type III, approximately 17 and 4 molecules per cell, respectively), where these foci are missing. Similarly, GAS5 has dominant nuclear foci in HeLa cells (type II, approximately 195 molecules per cell), and less frequent foci in fibroblasts, where its expression is lower (type IV, approximately 75 molecules per cell). In other cases, higher abundance was associated with the appearance of RNA in the cytoplasm as well. For example, lincFOXF1 was more abundant in fibroblasts than in HeLa cells, where it more frequently appears in the cytoplasm (type IV in fibroblasts vs. type II in HeLa cells; Additional file 1: Figure S8).

We next applied single molecule RNA FISH for a few of our lncRNAs on tissue sections [57,58] to test whether the localization patterns we observed in cultured cells were consistent with the patterns found in intact tissues. We selected MALAT1, NEAT1, and PVT1 (XLOC_006922), which have orthologous expressed transcripts in mouse, and performed single molecule RNA-FISH in both mouse embryonic stem cells (mESCs) and mouse neonatal cardiac/kidney tissue (Methods). For each of these lncRNAs, we observe the same unique focal nuclear pattern across species (that is, in both HeLa cells and mESCs) and in the mouse tissue (Additional file 1: Figure S10; Methods), showing that the patterns we observed in cultured cells recapitulate what we observed *in vivo*.

### lncRNAs do not persist at nuclear foci during mitosis

The appearance of bright nuclear foci of specific lncRNAs raised the question of whether these foci persist through mitosis; persistence at the target locus through mitosis could suggest that lncRNA play a role in potential mechanisms for the maintenance of epigenetic states through cell division. To address this question, we examined the staining in mitotic cells of six lncRNA that exhibit nuclear specific localization patterns (approximately 50% of such cases).

None of the lncRNA we examined exhibited nuclear foci in cells undergoing mitosis (Figure 3e; Additional file 5). (The potential foci we observed in approximately one-third of ANRIL mitotic cells were not validated when using two-color co-localization; Additional file 5). Notably, for five of the lncRNAs, including XIST, we observed some molecules spread throughout the cytoplasm during mitosis (consistent with previous observations for XIST [6]). In the case of XLOC_001515 we did not observe any lncRNA molecules whatsoever during mitosis. Thus, we found no evidence for mitotic retention of these lncRNA to the nuclear foci they inhabit during interphase.

### The extent of cell-to-cell variability in lncRNA expression is similar to that of mRNAs

When measured in bulk cell populations, lncRNAs are typically expressed at low levels compared to mRNAs [4,9]. Several studies have hypothesized that these bulk measurements may obscure an extreme cell-to-cell heterogeneity in which lncRNA are expressed very highly in a small fraction of cells, but lowly or not at all in most others cells, resulting in average low expression [10,59]. We tested this hypothesis by quantifying the cell-to-cell variability of the lncRNAs in our panel.

**Figure 4:**
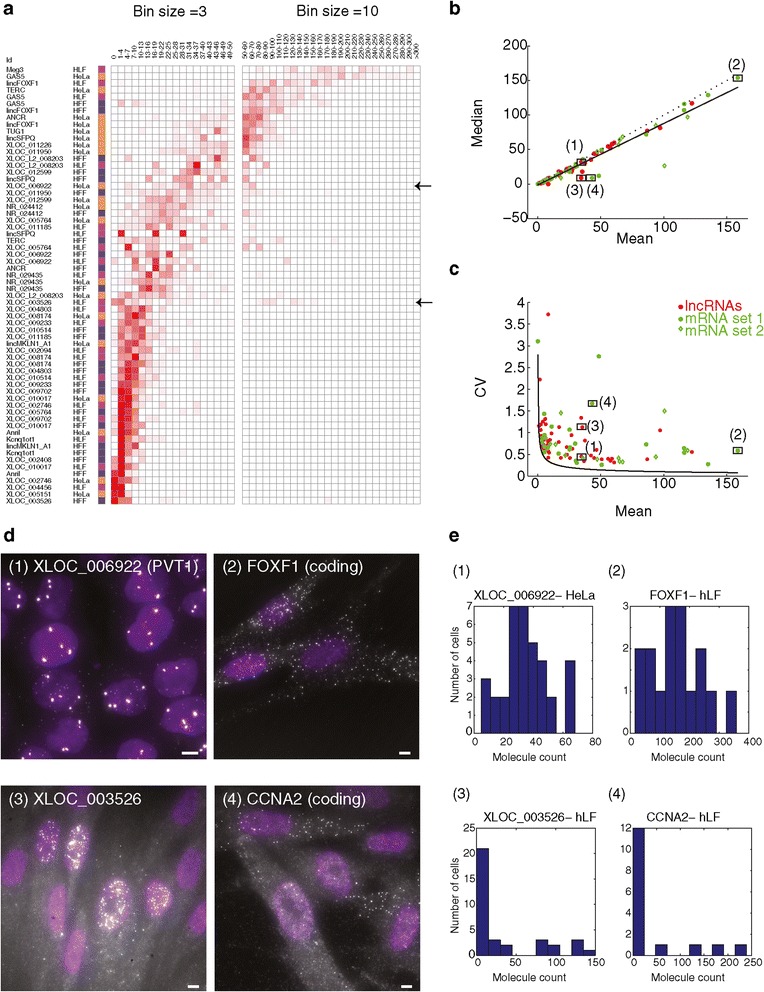
**Cell-to-cell variability does not appear to explain the low abundance of the lncRNAs in our survey. (a)** Distribution of RNA single molecules counts (bins, columns; Red intensity: fractional density of molecule counts across the population) for the 64 lncRNA-cell pairs in the validated set that are quantitative (rows, Methods). Cell type color coding: orange - HeLa, blue - hFF, purple - hLF. Left bins are sized 3 (0 to 50 molecules ), where right in bins are sized 10 (50 to 300 molecules). A heterogeneously expressed lncRNA (XLOC_003526) and a homogenously expressed lncRNA (XLOC_006922), are pointed by black arrows and referenced in figures b and c. **(b, c)** The relationship between the mean molecule count (X axis) vs. median molecule count (Y axis, b) or vs. variability in molecule counts (Y axis, coefficient of variation, c) for the 64 lncRNA-cell pairs in the quantitative validated set (red), mRNA set 1 (green circles; Methods) and mRNA set 2 (green diamonds; Methods). A linear regression line in b (black) supports the consistency of the majority of transcript-cell pairs with a unimodal distribution (Y = 0.87X-1.25, Pearson r = 0.96). Dotted line is Y = X. Black curve in (c) is the theoretic Poisson distribution. Four transcripts marked (1 to 4) are analyzed further in d and e. LncRNA pairs with mean >170 (less than 10% of all pairs) are not presented, but show a similar pattern on a log scale. **(d)** Fluorescence micrographs of single molecule RNA FISH of a homogenously expressed lncRNA (1-XLOC_006922; top left) and mRNA (2-FOXF1; top right) and of a heterogeneously expressed lncRNA (3- XLOC_003526; bottom left) and mRNA (4 - CCNA2; bottom right). XLOC_003526 and CCNA2 are both heterogeneous but do not correlate with each other based on co-staining in two colors. Scale bar, 5 μm. **(e)** Molecule count distributions for each of the example transcript 1 to 4.

We first confirmed that the average (cell population) expression level estimates for our lncRNAs were generally consistent between RNA FISH and RNA-Seq (Pearson r = 0.55; *P* value <2.5×10^-6^; Additional file 1), with discrepancies possibly due to the high variability in RNA-Seq abundance estimates for some of the examined transcripts (Additional file 1: Figure S11). We observed even higher consistency with qPCR (Pearson r = 0.788, *P* value <3.96×10^-3^, in comparison to Pearson r = 0.579 when comparing RNA-Seq on the same subset of genes; Additional file 1: Figure S12; Methods), as also reported by others [44-48].The distribution of single cell counts demonstrated the relatively low overall expression of lncRNAs, with 43% of lncRNA-cell pairs having 10 or fewer molecules per cell on average and with a median of 14 molecules across all gene-cell-pair distribution medians (vs. 36 for the 49 mRNA-cell pairs we examined) (Figure 4a).

We also checked whether any of our lncRNAs showed evidence for G1 or S/G2/M dependent expression by simultaneously measuring the cyclin CCNA2 transcript count in every image we obtained, which is high in the S, G2, and M phases of the cell cycle [60,61]. We identified two lncRNAs whose expression positively correlated with CCNA2 (lincSFPQ and XLOC_001226), and one negatively correlated (XLOC_011185), (Additional file 5; Additional file 1: Figure S13), suggesting that expression of these lncRNAs was regulated through the cell cycle. Still, for the majority, any variability we observed was not due to variability in cell cycle phase.

In most cases, cell-to-cell variability in lncRNA levels was similar to that of protein coding mRNAs expressed at comparable average levels and did not reveal the presence of low frequency, highly expressing cells (Additional file 1; Figure 4c). In particular, the mean and the median molecule counts were similar, highlighting the lack of outlier cells in the single cell distributions (Additional file 1: Methods; Figure 4b; Additional file 1: Figure S9; Pearson r = 0.98, *P* value <2.5×10^-39^). One notable exception was the tissue specific lncRNA XLOC_003526 encoded from a poorly conserved 900 Kb gene desert (Figure 4d, e): it is lowly expressed on average (FPKM <1 in a population of hLF RNA-Seq, with few, if any, spliced reads; Additional file 1: Figure S14), but in RNA-FISH approximately 25% of the cells express it highly (107 +/- 26 molecules on average), whereas the other cells express it very lowly (9 +/- 1.2 molecules on average). Its expression did not correlate with CCNA2, suggesting that its variability is not related to cell cycle.

Since we only obtained a few dozen cells for most of the lncRNA-cell line pairs examined (due to limited imaging throughput), we could not rule out the possibility of a particularly rare cell with extraordinarily high expression levels. To increase our statistical power, we imaged 500 to 700 cells for each of four lncRNA in HeLa cells (Additional file 1: Figure S15), including XLOC_004456, which displayed no signal in HeLa in our initial assessment. None of these images revealed the presence of any highly expressing outlier cells. With a sample size of n = 500 cells, we can place an upper bound of 0.6% of cells that may express high levels of the lncRNA but went undetected in our assay with a statistical power of 0.95 (Additional file 1).

### Cellular localization and expression correlation of divergently transcribed lncRNA-mRNA transcript pairs

**Figure 5:**
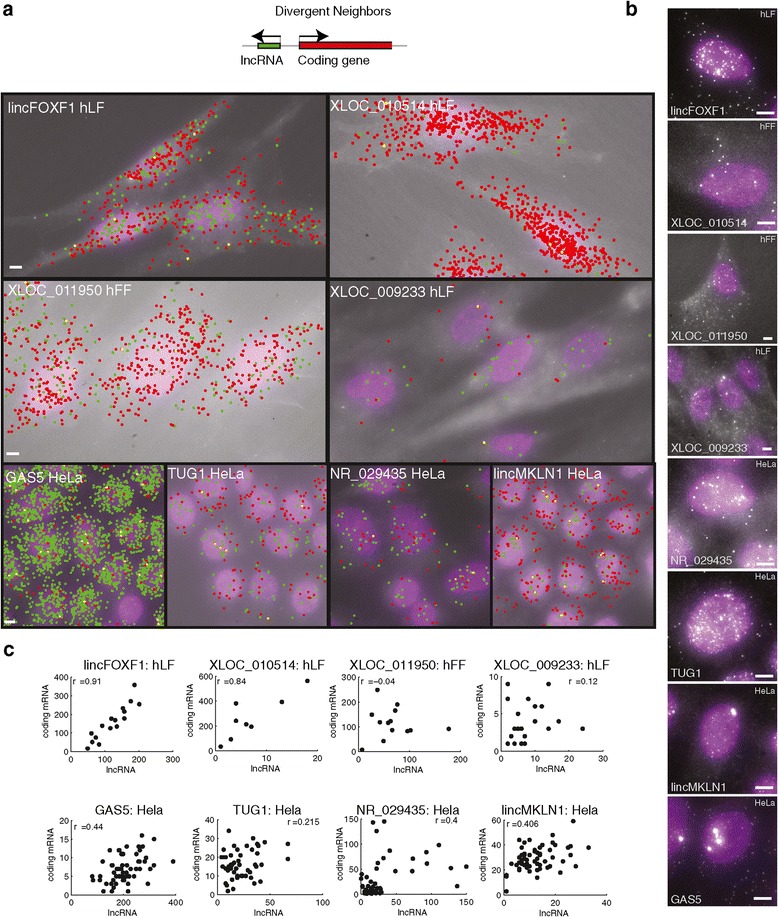
**Cellular localization of divergent lincRNAs and their neighbors. (a)** Two-color overlay micrograph presenting florescence probes targeting the lincRNA (green) and coding neighbor (red). Co-localized spots are marked yellow. The lincRNA and cell type are marked on the image. Scale bar, 5 μm; marked on the left most image. Top: illustration of the positional genomic orientation of a divergent lincRNA and its coding gene neighbor. **(b)** Representative fluorescence micrographs as shown in Figure 2 for the lincRNAs in a. Scale bar, 5 μm. **(c)** Scatter plots of the relationship in each cell between the expression level of the lincRNA (X axis, molecule count) and that of its neighboring coding gene (Y axis). Pearson correlation coefficients (r) after removal of outliers (Additional file 1) are denoted on top. Data in **(a-c)** are presented for eight of the nine lincRNA-gene neighbor pairs for which a valid probe set exists.

We have previously distinguished a subset of lincRNAs that are transcribed divergently from protein coding genes’ promoters (approximately 500, approximately 13% of human lincRNAs [9,35]; Figure 5a), but are stable, processed and spliced. One hypothesis is that these ‘divergent’ lncRNAs are co-regulated with their neighbors and possibly have a regulatory effect on their neighbor at the transcription site [35,62], with bulk assays observing co-expression of divergent transcripts [35,42,43,62]. To look for correlations at the single cell level and potential localization to the site of transcription, we simultaneously measured abundance and localization of divergent lncRNA and their mRNA neighbor for eight of the nine candidate divergent lncRNAs for which we had valid probe sets (Figure 5; Additional file 5).

We observed that in most cases (7/8) the bi-directionally promoted lncRNAs were not simply localized at one or few foci (characteristics of type I; likely to be the site of transcription), but rather were located throughout the cell (Figure 5a and b; Additional file 1: Figure S16). For example, RNA from XLOC_011950 and XLOC_010514 were substantially cytoplasmic and showed no nuclear foci (type VI). NR_029435, TUG1, and XLOC_009233 RNA were mostly nuclear but with no apparent foci (type III). Lastly, lincMKLN1 (type II; also known as PINT [63]), lincFOXF1 (also known as FENDRR [64]), and GAS5 (type II and VI) RNA were all present as nuclear foci in some cell types. Substantial numbers of lincFOXF1and GAS5 RNA were also found outside these foci and in the cytoplasm. Together, the subcellular localizations displayed by divergent lncRNAs were distinct from each other, and were not qualitatively different from those of the other lncRNAs in our survey.

We also observed a spectrum of correlation and expression levels of the lncRNA and its neighboring protein coding gene (Figure 5c). Both lincFOXF1 and XLOC_010514 tightly correlated with their neighbors in hLFs (Pearson r = 0.91, 0.84, respectively). XLOC_011950 and its neighbor are positively correlated in HeLa cells, but did not correlate in hFFs, where they were still expressed to the same extent on average (Figure 5c; Additional file 1: Figure S17). NR_029435 and GAS5 were positively correlated with their neighbors in HeLa cells (Pearson r = 0.4 and 0.44, respectively), although it is possible that these relatively mild correlations resulted from a generic correlation with cellular volume (Padovan-Merhar and Raj, personal communication). We note that there was no correspondence between the existence of an expression correlation between the lncRNA and its neighbor and a particular subcellular localization pattern. Taken together, while the divergent lncRNA in this study shared a common genomic layout, no consistent pattern of localization nor co-expression levels with their neighboring coding gene emerged.

## Discussion

In this study, we applied single molecule RNA FISH to quantitatively characterize the expression and localization of 34 lncRNAs chosen to span diverse characteristics at the single cell and subcellular level in three human cell types (overall, 70 gene-cell pairs). Our analysis provides a quantitative framework, important controls, and considerations for analyzing fundamental properties of lncRNAs by RNA FISH. Using this approach, we have shown that lncRNAs’ localization patterns are formed of combinations of a set of archetypical localizations, including a variety of predominantly nuclear localization patterns. These patterns suggest the possibility that these particular localizations correspond to functional categories. We also found that they express in a mostly uniform manner from cell to cell, and do not remain attached to chromosomes during mitosis.

While single molecule RNA FISH has the potential to be a very powerful technique for the analysis of lncRNA, our results emphasize that one must exercise extra caution in this application of the technology. We found that the background resulting from one ‘rogue’ oligonucleotide binding off target can often resemble legitimate lncRNA signal patterns, such as nuclear foci. For an mRNA, typically, the vast majority of the RNA is cytoplasmic; thus, counting any suspect nuclear foci will not greatly affect the overall quantification. However, for many legitimate lncRNAs, it is precisely this sort of nuclear staining pattern that may be of interest, making it difficult to ignore such signals. In general, we have not found particular rules for which oligonucleotides lead to this background, and hopefully future bioinformatics algorithms can limit these issues, perhaps by further refining strategies to avoid repetitive elements which may be transcribed at high levels. Regardless, our extensive troubleshooting and validation strategies strongly suggest that two-color validation of lncRNA FISH probe sets is crucial to ensuring the validity of RNA FISH signals.

Overall, we observed a strong bias towards nuclear localization of lncRNA, with 95% of them having a higher nuclear fraction than mRNA. Beyond that, our technique also afforded sufficient spatial resolution to distinguish different subnuclear patterns. (The cytoplasmic lncRNA we observed did not show any readily discernable patterns.) One commonly observed pattern was bright, tightly localized nuclear foci (approximately 30% of our set), which may be consistent with a role for these lncRNAs in chromatin regulation [5], as shown for XIST [15], KCNQOT1 [51], AIR [65], and other lncRNA involved in imprinting [66]. These were likely localized to the transcription site itself, potentially during transcriptional bursts [67], and did not persist during mitosis.

The pattern we observed for MEG3 was one reminiscent of MALAT1, which is known to localize to nuclear speckles and was shown to affect various cellular processes [16]. This pattern was almost solely nuclear, and showed a ‘clumping’ that may indicate association with specific nuclear bodies [68]. MEG3 is an imprinted lncRNA which is downregulated in many types of cancers and previously hypothesized to function as a tumor suppressor in a mechanism that is still not well understood [69-71]. Interestingly, co-staining for these two lncRNA showed that a substantial and significant fraction of MEG3 molecules co-localized with MALAT1 (Additional file 1: Figure S6). These results suggest the possibility that MEG3 and MALAT1 are functionally related, showing the potential for our image-based approach to reveal relationships between lncRNAs that would not be apparent through other methods.

Our single cell analysis suggests that - at least for the set we examined - the low abundance of lncRNAs in bulk cell population was most likely not a result of high expression in a small subset of ‘jackpot’ cells as previously hypothesized [10,59]. Overall, the extent of cell-to-cell variability of lncRNAs resembled that of mRNA expressed at similar levels. Although in some cases the number of imaged cells is low, we nevertheless observe a relatively homogenous expression of few molecules per cell (Figure 4). This conclusion is bolstered by our analysis of over 500 cells for a few representative lncRNAs. Some lncRNA (notably XLOC_003562, expressed at approximately 110 molecules per cell in approximately 25% of the cells) display high levels of variability, but this is within the range of variability also observed for mRNAs, and the frequency of positive cells was not particularly low. We cannot definitively rule out the possibility that very rare ‘jackpot’ cells exist, but they are not necessary to explain the average expression in bulk assays. One interesting observation, however, was two rare daughter cells, probably resulting from asymmetric division of HeLa cells, which contain high levels of NR_029435 (Additional file 1: Figure S18). It is hard to know if this finding has biological meaning, or was just a symptom of cytological abnormalities in cultured HeLa cells.

While almost all divergent transcription results in short unstable transcripts [41-43,72,73], we and others have reported over 500 lincRNAs that are transcribed divergently to protein coding genes [9,35,62]. We examined eight of these pairs in detail, wondering if they exhibited any features that may distinguish this class of lncRNA. We found a variety of characteristics, with varied abundances and localizations ranging from almost exclusive nuclear foci to broadly cytoplasmic. Moreover, correlations with the neighboring genes revealed some potential regulatory interactions for a few of the lncRNA in our set, but no general rule emerged; indeed, a recent model suggests that divergent transcription may be a mechanism for evolving new, functionally unrelated genes [74] rather than signifying a regulatory mechanism *per se*. Overall, our results suggest that these lncRNA may have a variety of functions despite their common genomic layout.

## Conclusions

Collectively, our study highlights important differences and similarities between lncRNAs and mRNAs, including a characterization of the subcellular localization of lncRNAs. This study further provides a workflow for applying single molecule RNA FISH to study lncRNA. The rich set of localization patterns we observe suggest a broad range of potential functions for lncRNA and highlights specific lncRNAs for future mechanistic studies.

## Methods

### Design and synthesis of RNA FISH probe sets

We designed oligonucleotides sets using software available through Stellaris Probe Designer [75]. Since the software avoids sequence elements deemed to cause high levels of background, it can sometimes result in only a limited number of potential oligonucleotides targeting a particular RNA. As a conservative choice, we only included in the actual screen those lncRNAs for which we had at least 10 designed oligonucleotides. Additional file 3 contains all the oligonucleotide sequences used in this study.

We ordered all Stellaris™-type oligonucleotides from Biosearch Technologies, but instead of a dye on the 3′ end of the oligonucleotide, we ordered oligonucleotides with an amine group on the 3′ end, to which we coupled either Alexa Fluor 594 (Life Technologies), Cy3 (GE Healthcare) or Atto 647 N (Atto-Tec). After coupling, we removed the unlabeled oligonucleotides via HPLC purification. For the data using full probe sets, we labeled the lncRNA oligonucleotides with Alexa Fluor 594, the coding neighbor mRNA oligonucleotides (when applicable) with Cy3, and Cyclin A2 mRNA oligonucleotides with Atto 647 N. When validating the lncRNA oligonucleotides via co-localization, we labeled the even numbered oligonucleotides in Alexa Fluor 594 and the odd numbered oligonucleotides with Cy3.

### Cell culture, tissue collection, and RNA FISH

We cultured human foreskin fibroblasts (CRL-2097, ATCC), human lung fibroblasts (IMR-90, ATCC), and HeLa cells (gift from the lab of Phillip Sharp, MIT) in Dulbecco’s modified Eagle’s medium with Glutamax (DMEM, Life Technologies), supplemented with 10% fetal bovine serum, Penicillin and Streptomycin. We grew the cells in 2-well chambered coverglass (Lab Tek). We washed cells with 1x phosphate buffered saline (PBS) and then fixed them in 3.7% formaldehyde in 1X PBS for 10 min at room temperature. After fixation, we washed the cells twice with 1X PBS and then permeabilized them in 70% ethanol at 4°C at least overnight or until we performed RNA FISH staining.

We collected tissue sections following a modified version of the protocols described in [57,58]. Briefly, tissue harvested from neonatal mice was immediately flash-frozen in OCT (optimal cutting temperature compound) in liquid nitrogen. We stored frozen tissue blocks at -80°C prior to sectioning. Five micron thick sections were cut at -20°C and adhered to positively charged slides. Immediately after sectioning, we washed tissue sections briefly with 1X PBS and fixed in 3.7% formaldehyde for 10 min. Following fixation, we washed twice with 1X PBS and then submerged slides in 70% ethanol for permeabilization and storage of tissue at 4°C until performing RNA FISH.

We performed RNA FISH staining as previously described [24,76]. Briefly, we washed cells with a solution of 10% formamide in 2X sodium citrate buffer (SSC), then applied the appropriate amount of probe in a hybridization solution containing 10% formamide, 2X SSC, and 10% dextran sulfate (w/v). Hybridization was allowed to occur overnight in a humid chamber at 37°C. Cells were then washed twice for 30 min at 37°C with 10% formamide in 2X SSC. DAPI was applied during the second wash. Cells were then rinsed twice with 2X SSC before imaging.

### Imaging

After performing RNA FISH, we imaged the cells on a Nikon Ti-E inverted fluorescence microscope using a Plan Apochromat 100X objective and a cooled CCD camera. We acquired around 25 to 30 optical slices at 0.3 μm intervals, thereby covering the entire vertical extent of the cell. As described previously, we used bandpass filters specifically for these channels that have essentially no signal crossover [61], and acquired successive image stacks for DAPI (nuclear stain), each fluorescence channel targeted with an RNA FISH probe. We also acquired images in a fluorescence channel with a 488 nm excitation (similar to fluorescein/Alexa 488); this channel has no probe in it, and thus reveals the degree of autofluorescent background in the sample.

### Image analysis

Image analysis was performed using custom software written in Matlab (The Mathworks, Natick, MA, USA) as previously described [24]. Briefly, images were first manually segmented to define cellular boundaries by using a custom user interface. Images were then processed with a linear filter akin to a Laplacian-of-Gaussian to remove non-uniform background and to enhance particulate signals. RNA particles in each channel were then identified in a semi-automated manner by selecting an intensity threshold above which a spot is considered an RNA particle. Specifically, the threshold was computationally estimated (and then manually confirmed or adjusted) by identifying a plateau in the graph comparing the intensity threshold (X axis) and total particles above that threshold (Y axis; Additional file 1: Figure S19). The accuracy of this threshold may vary from RNA to RNA depending on the quality of the signal, but we generally believe that our spot detection algorithms are typically accurate to within 10% [67] for the following reasons. First, our numbers match well with absolute RT-qPCR [44-47]. Second, when we label two parts of the same RNA molecule with different colors and then look for co-localization, we typically see very strong co-localization of roughly 95% or more [48,77]. We then determined each spot’s intensity by fitting a two-dimensional Gaussian to the spot signal and obtaining amplitude. Finally, we determined which spots co-localize across channels following the methods outlined in Levesque *et al.* [77] in a two stage process: first, we find spots that co-localize within a relatively large spatial window, then we use those co-localized spots to register the two images (correcting for any shifts between channels) and run the co-localization again, but this time with a smaller window. We ignored spots that co-localized with spots identified in the GFP channel (which represent auto-fluorescent background). Details regarding subsequent analysis steps are described in the following sections.

### Validation of probe sets by two-color co-localization

To validate each probe set we used a two-color co-localization approach similar to that previously described [23,24]. Briefly, we partitioned each probe set to the even- and odd- numbered oligonucleotides and coupled each subset with a different fluorophore (evens with Alexa 594, odds with Cy3). We then hybridized the two probe sets and imaged each color.

To determine the total number of RNA particles above background signal in each color we pursued the following procedure. First, we determined the total number of particles imaged in each cell using the full probe set coupled to Alexa 594 (termed the ‘single-colored probe set’), using the previously described, semi-automated procedure [24] employed in Image Analysis, above (Additional file 1: Figure S19). We also estimated the distribution of particle counts for the single-colored probe set and its mean *m*_*i*_. Next, for every cell in the two-color co-localization dataset we selected the *x*_*i*_ particles with the highest signal for each of the even-numbered and odd-numbered probe subsets, where *x*_*i*_ *= max (50*, 5*m_*i*_*)*. We then calculated the number of co-localized spots among these *x*_*i*_ spots from each color in every cell. Finally, we determined the distribution of the number of co-localized spots for each probe set across cells. We only consider the co-localized spots as representing a true mRNA particle in each channel when we analyze images acquired in the two-color assay.

We applied this analysis to every probe set in each of the three cell types (HeLa, hLF, hFF) in which it displayed a signal. A probe set was considered invalid in a specific cell type if there was either (Figure 1a, Additional file 1: Figure S2d): (1) a qualitative difference between the localization pattern obtained using one color channel vs. the other; or (2) a quantitative difference defined as a statistically significant difference in the distribution of the number of co-localized particles and the single-color probe set particles (*P* <0.05, Mann-Whitney U rank sum test). The remaining cell-probe set pairs were considered valid and images acquired with the full-single-colored probe set were used for all subsequent analyses. Manual examination recovered 14 additional borderline cases in which the clear pattern seen in one cell type was similar to that in a different cell type for which the two color and single color assays were consistent. The specific classifications and distribution comparisons are specified in Additional file 4 and Additional file 1: Figure S21.

For many of the two-color experiments it was impossible to robustly determine the total number of mRNA particles in each channel using the plateau method [24] used for the single-colored probe set (Additional file 1: Figure S19b). This is likely due to the smaller number of oligonucleotides that actually hybridize to the target when using only half the probe set, resulting in a lower contrast between the real signal and background [24]. The approach we used above to evaluate the number of co-localized spots does not rely on the plateau method and is not sensitive to the selection of an intensity threshold.

### Localization to the nucleus

Nuclear localization of a spot was heuristically determined based on co-localization with DAPI after considering the maximal signal across all z-stacks. We determined nuclear localization by two approaches that yielded similar results: (1) the percent of spots across the entire cell population localized to the nucleus (‘molecule level’); or (2) the percent of cells in which more than 50% of the spots were localized to the nucleus (‘cell level’). Classification of a gene as predominantly nuclear was estimated based on the ‘cell level’ approach by calculating the fraction of nuclear spots for each cell, and then taking the median across this distribution.

Each lncRNA:cell-type pair was assigned to one of the following classes: (I) one or two large foci; (II) both large foci and single molecules scattered through the nucleus; (III) predominantly nuclear (without foci); (VI) cytoplasmic and nuclear; and (V) predominantly cytoplasmic.

Assignment was performed with the following steps: (1) For each lncRNA-cell pair we calculated the fraction of nuclear spots for each cell, and then determined the median of that distribution. (2) LncRNA-cell pairs with a median fraction of nuclear spots >0.65 were then manually assigned to classes I, II, or III, by manual inspection of the images and visual recognition of large foci. (3) LncRNA-cell pairs with a median fraction of nuclear spots <0.35 and an average spot count >20 were classified as V. The selection of a spot count threshold was made in order to be conservative when classifying to V. (4) All other cases were classified as IV. (5) Finally, we reassigned two borderline cases to IV (lincFOXf1-hFF and XLOC_011950-hFF, median nuclear fraction of 0.67, 0.35 respectively), since we were unable to manually identify specific cells that support a predominant localization to either compartment. Assignments to localization patterns are specified at Additional file 5.

### RT-qPCR

We performed RT-qPCR on subset of lncRNAs in our set spanning a broad range of expression in HeLa for which we were able to design qPCR primers with high efficiency (>85%) (Additional file 3; three biological replicates). We used these data to compare RT-qPCR expression estimates and RNA FISH molecule counts.

Total RNA from HeLa cells (three biological replicates) was isolated using RNeasy mini kit (Qiagen, Venlo, Netherlands) according to the manufacturer instructions. cDNA was generated using SuperScript III First-Strand Synthesis System for RT-PCR (Invitrogen) kit and RT-qPCR was performed using FastStart Universal SYBR Green Master (Roche) according to the manufacturer instructions on a 7900HT Fast Real-Time PCR System (Applied Biosystems).

### Catalog access

Our lncRNA FISH catalog can be accessed at [78] (select lincRNA-FISH catalog on the left menu). All supplementary datasets as well as raw image data can be downloaded from the website. Individual images can be viewed through an image database linked to the website.

### Accession number

RNA-Seq data are available through GEO, GSE57049.

## Additional files

Additional file 1:
**Supplementary text and figures.**
Additional file 2:
** Supplementary dataset 2, RNA-Seq analysis.**
Additional file 3:
**Supplementary dataset 3, candidate set info.**
Additional file 4:
**Supplementary dataset 4, two-color validation analysis.**
Additional file 5:
**Supplementary dataset 5, single cell analysis of valid set.**

## Abbreviations

hFF: human foreskin fibroblasts
hLF: human lung fibroblasts
lincRNAs: large intergenic non-coding RNAs
lncRNAs: long non-coding RNAs
RNA FISH: RNA fluorescence *in situ* hybridization
RNA-Seq: RNA sequencing
