# Supplementary material for: Localization and abundance analysis of human lncRNAs at single-cell and single-molecule resolution
Source: Genome Biol. 2015 Jan 29;16(1):20. doi: 10.1186/s13059-015-0586-4 (PMC4369099; doi:10.1186/s13059-015-0586-4)
Supplement: Additional file 1: — Supplementary text and figures. [file 13059_2015_586_MOESM1_ESM.docx]

**Localization and Abundance Analysis of Human lncRNAs at Single Cell and Single Molecule Resolution**

Moran N. Cabili^1,2,3,*^†, Margaret C. Dunagin ^4,*^, Patrick D. McClanahan^4^, Andrew Biaesch^4^, Olivia Padovan-Merhar^4^, Aviv Regev^1,5, §^, John L. Rinn^1,3, §^† and Arjun Raj ^4, §^†

^1^ Broad Institute of MIT and Harvard, 7 Cambridge Center, Cambridge MA, 02142, USA

^2^ Department of Systems Biology, Harvard Medical School, Boston, MA, 02115, USA

^3^ Department of Stem Cell and Regenerative Biology, Harvard University, Cambridge, MA, 02138, USA

^4^ School of Engineering and Applied Science, University of Pennsylvania, Philadelphia, PA, 19104,USA

^5^ Howard Hughes Medical Institute, Department of Biology, Massachusetts Institute of Technology, Cambridge, MA, 02140, USA

^*^ Equal contributors

^§^ Equal contributors

† Corresponding authors

Email addresses:

MNC: nmcabili@ broadinstitute.org

MCD: dunagin@seas.upenn.edu

PDM: pmccl@seas.upenn.edu

AB: biaescha@gmail.com

OPM: opadovan@sas.upenn.edu

ARegev: aregev@broadinstitute.org

JLR: john_rinn@harvard.edu

ARaj: arjunraj@seas.upenn.edu

**Supplementary Material**

**Supplementary Methods**

**Selection of candidate lncRNA set**

We manually selected the lncRNAs candidate set from our human lincRNA catalog [[1](#_ENREF_1)] to represent different features: (**1**) tissue specificity, (**2**) evolutionary conservation by an expressed mouse syntenic ortholog, and (**3**) divergent transcription from coding genes’ promoters. We selected the set such that it can be divided to a positive and a negative subset with respect to each trait so we can study teach feature with respect to other features (*e.g.*, expression level, nuclear localization, etc.). These subsets were not mutually exclusive across these three features (**Figure 1b**). We also ensured that the set includes transcripts expressed in at least one of the three cell types we studied (hLF, hFF or HeLa), and represent a wide range of expression levels (as we did not have an estimate for the minimal expression level required for detection by RNA-FISH).

Features of each lncRNA were extracted from our human lincRNA catalog and were evaluated as previously described [[1](#_ENREF_1)]. Briefly, to evaluate tissue specificity we use a tissue specificity score (ranging from 0 to 1) that is calculated by an entropy based metric. We consider transcripts with a score greater than 0.4 as tissue specific. Divergent lncRNAs are those that are bi-directionally transcribed within 10 KB from a protein-coding gene promoter (illustrated in **Figure 1a**). Orthologous lncRNAs in this study are those for which there is an annotated expressed transcript in their syntenically mapped genomic region in mouse (mapped by the TransMap algorithm [[2](#_ENREF_2)]).

The selection process was performed by the following steps. **(1)** we selected only the lincRNAs that were is the stringent set and were significantly expressed in foreskin or lung fibroblasts. We determined significant expression based on a scan statistic [[1](#_ENREF_1), [3](#_ENREF_3)]. **(2)** We divided the set from (1) to two subsets according to the presence of a mouse syntenic ortholog. **(3)** We sorted each of the two subsets by *(a)* expression level, *(b)* tissue specificity, *(c)* transcript length, *(d)* the presence of a chromatin signature of actively transcribed genes [[1](#_ENREF_1), [3](#_ENREF_3)], and *(e)* being a divergent transcript. **(3)** We then screened the sorted subsets top-down manually selecting those lincRNAs that had an isoform that was clearly supported by RNA-Seq spliced read based on visual inspection of the RNA-Seq read alignments and the reconstructed transcripts. **(4)** We partitioned these candidates to three expression bins and balanced the subsets sizes by eliminating transcripts from the top two bins, giving preference to divergent transcripts, tissue specific transcripts, and then to longer transcripts that have a chromatin signature of actively transcribed genes and a low number of isoforms. **(5)** We repeated steps 1-4 using the transcripts expressed in HeLa cells. All selection criteria were based on previously estimated features that are available through the human lincRNA catalog table ( [[1](#_ENREF_1)] ; http://www.broadinstitute.org/genome_bio/human_lincrnas/). Finally, we included 16 previously studied lncRNAs curated from the literature (**Additional file: 2.3**).

**Selection of control mRNA sets**

We selected two sets of mRNA controls (**Additional file: 2.4-2.5)**. Set 1 is comprised of mRNAs transcribed divergently to our ‘divergent lncRNAs’ and CCNA2, a cyclin simultaneously imaged in all images. Set two consists of mRNAs selected to span a wide range of expression levels in human foreskin fibroblasts (Padovan-Merhar and Raj, personal communication).

**Single molecule count correction**

We applied the following heuristic to estimate the number of molecules within a large focus. For each lncRNA, we divided the total integrated signal in a single detectable spot (focus) by the median signal of the spots within the same cell. The final spot count *x_i_* for a spot *p_i_*  was *x_i_ = max (1, floor(s_i_/c_i_))*, where *s_i_* is the total intensity of spot *p_i_*, and *c_i_* is the median intensity of across all the spots within the same cell as *p_i_*. *c_i_* was used to estimate the signal intensity from hybridization to a single molecule. Application of this correction affected on average 5% +/- 0.5% STDV of the originally-detected spots, causing on average an increase of 12% +/- 1.7% in the mean spot counts (**Figure S20**). Following this calculation, the spot count estimations of XIST, MALAT1, and NEAT1 were outliers in that the corrected counts resulted in over an order of magnitude higher estimate than the other cases. Moreover, shorter exposure time was required in these three cases in order to obtain a clear image, as molecules in these cases are clumped together posing a challenge to resolve single spots. Therefore, we consider the spot count estimation of XIST, MALAT1, and NEAT1 as not quantitative and eliminate these from the quantitative parts of the analysis. Five other lncRNAs (Anril, GAS5, NR_029435, lincFOXF1, and TERC) included outlier cells (<4 cells per lncRNA) for which the fitting algorithm yielded an implausibly large value for a specific spot, as verified by eye (resulting in a total spot count a 15 IQR (inter quartile range) higher than the median count across all imaged cells for that gene). These specific cells were eliminated from the analysis (visual inspection confirmed these were not “jackpot cells”; **Additional file: 3.4**).

**Identification of off-target hybridization of an XLOC_010514 probe to MALAT1**

To identify candidate oligonucleotides of the XLOC_010514 lncRNA that potentially hybridize to other RNAs in the cell we aligned the sequences against the Refseq [[4](#_ENREF_4)] transcriptome using BLAST [[5](#_ENREF_5)] (with default parameters for short read alignments; word size =7, match score =1 mismatch score = -3). We then ranked the next best hits based on their expression levels by RNA-Seq. Elimination of oligonucleotide #5 that is predicted to have 15 exact matches with MALAT1 and reimaging resulted with elimination of the localization pattern that was similar to MALAT1.

**Estimation of population expression abundance based on RNA-Seq**

We estimated the expression abundance of all lncRNAs and protein-coding genes by running Cuffdiff2 (non-diff mode) [[6](#_ENREF_6)] across a set of HeLa, hLF and hFF samples as well as a second set that included these samples in addition to a previously published RNA-Seq human tissue compendium ( [[1](#_ENREF_1), [7](#_ENREF_7)]; **Additional file: 5**). RNA-Seq libraries were aligned to Hg19 using Tophat [[8](#_ENREF_8)]. We used our entire human noncoding transcripts catalog [[1](#_ENREF_1)] complemented with additional lncRNAs added in this screen and all coding transcripts annotated in the UCSC Browser [[9](#_ENREF_9)] for a comprehensive representation of transcripts along the genome, while performing abundance estimation (**Additional file: 5**). FPKM calls were log_2_-transformed (after addition of ε = 0.05). Expression matrices and other information related to the RNA-Seq analysis are provided in **Additional file: 5.**

**Identification of lncRNAs with extreme heterogeneity**

XLOC_003526 was first detected as having a subpopulation of highly expressing cells based on visual inspection of the data. We have attempted to identify other such cases systematically by two other approaches: (**1**) detecting outliers in the regression line fitting the samples’ mean and median. This detects only XLOC_003526. (**2**) Fitting a Poisson to the mean *vs*. CV plot and detecting outliers with a mean value outside the confidence intervals around the estimated mean. None of the candidates identified by the latter approach appeared to present a distinguished subset of expressing cells based on visual examination. Other attempts to fit a Gaussian or a mixture of Gaussians to model bimodality in the data failed due to small sample size.

**Selection of lncRNAs for a large sample size imaging survey**

We examined the lncRNAs that have a low average molecule count relative to their estimated expression based on RNA-Seq (by looking at off-diagonal data points in **Figure S11**) in HeLa cells (to enable higher throughput). We selected XLOC_L2_008203, XLOC_008174 and XLOC_004456, as they matched these criteria. We also included NR_029435 since we initially observed one rare case of a highly expressing cell and wanted to further estimate the frequency such case occurs.

**Rare cell power calculation**

We performed the following power calculation to determine the probability of not observing eve one ‘jackpot’ cell (defined informally as *max (100, 10*IQR(v)+mean(v))* , where *v* is the count vector and *IQR* is the inner quartile range) among *n* samples cells. Let *p* be the probability of a jackpot cell. Then the probability of not finding any jackpot cell among *n* sampled cell is (1-*p*)*^n^*. Therefore, the probability of finding at least one jackpot cell is 1-(1-*p*)^n^. In our studies, we measured at least n=500 cells without observing a jackpot cell. Setting our desired statistical power to 0.95, we achieve *p* < 0.006.

**Correlation with cell cycle phase**

To determine whether the expression of any of the lncRNAs in our study is correlated with phases of the cell cycle, we simultaneously measured in every image we acquired the cyclin CCNA2, which is exclusively expressed in the S, G2 and M phases of the cell cycle. We then applied several approaches to determine if a lncRNA’s expression is cell cycle dependent. (**1**) For every lncRNA – cell type pair we calculated the Pearson correlation coefficient (r) between CCNA2 and the lncRNA molecule counts. We consider |r|>0.4 as indication of cell cycle associated expression. For those associated cases, we estimate if the observed correlation was dependent on cell volume by fitting a linear regression model to predict the lncRNA molecule counts by using the cell area and CCNA2 levels as predictors (using the Matlab function LinearModel.fit). We then evaluated if CCNA2 was a significant predictor (P<0.05). (**2**) We estimated a threshold on CCNA2 levels for each cell type that distinguished cells in G1 from all other phases. We derived this threshold by plotting the distributions of levels of CCNA2 in all the cells we imaged from a specific cell type. The resulting distributions are bimodal, allowing us to determine a threshold t=0 for HeLa cells and t=20 for fibroblasts. For each lncRNA in every cell type we then split the population of cells by these thresholds and compared differences in the distributions of molecule counts using a KS test. Finally, we followed by a visual examination of the candidates that were significant based on these two criteria as a final conformation step.

**Divergent neighbor correlation analysis**

The presented Pearson correlation values between lncRNA-mRNA divergent pairs were calculated after removal of outliers. Outliers were defined as those that are three standard deviations over the mean of any one of the two variables. Correlation values before and after the removal of outliers are specified in **Additional file: 4.6**.

**Co-localization analysis of MEG3 and MALAT1**

To estimate the fraction of MEG3 molecule co-localize MALAT1 molecules, we defined the nuclear area covered by MALAT1 (determined as the area with intensity higher than the spot detection intensity threshold; **Methods**; **Figure S19**). These regions correspond to both the regions were MALAT1 molecules are clumped together (large foci) and spots with single molecules of MALAT1 and were manually examined. We then evaluate what fraction of the cells (hLF n=37 , hFF n=40) had a significant fraction of MEG3 co-localized with MALAT1. Significance was determined by a hypergeometric test after applying false discovery rate (FDR) correction (*1-hygecdf(x,M,K,N)*, where *x*: number of nuclear pixels covered by MALAT1 and MEG3, *M*:total nuclear pixels, *K*:total nuclear pixels covered by MALAT1, *N*:total nuclear pixels covered by MEG3).We also calculated the Pearson correlation coefficient of pixel intensities between the MALAT1 and MEG3 channels and determined a p-value for this correlation using random permutation. To do so we randomly shifted the MEG3 pixel intensity matrix ( *F(M,k) = [ M(k+1:n,:) ; M(1:k,:)]*, where *M* is a *n*m* matrix, and *k* is a random integer from *1:n* ) and recalculated Pearson correlation relative to the MALAT1 pixel intensity matrix, and thus derived a null distribution of correlation values.

**Supplementary Figures**


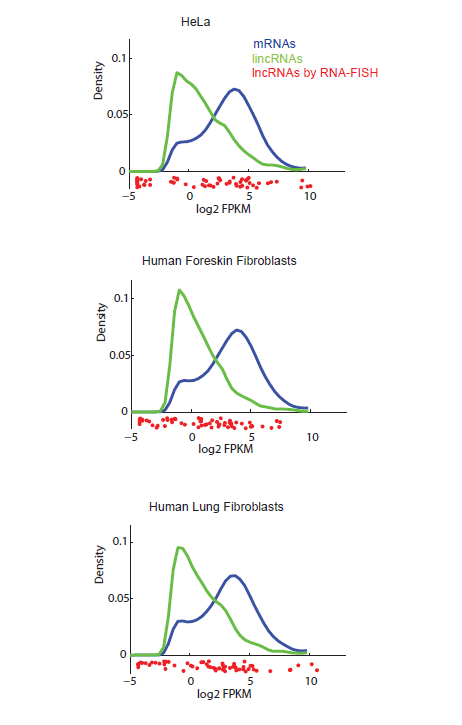


**Figure S1| LncRNA expression.** Shown are the distribution of (bulk) expression abundance estimates (log_2_(FPKM) as estimated by Cuffdiff2 [[6](#_ENREF_6)]) of all expressed lncRNAs (green), coding genes (blue) and the 61 selected lncRNAs for this study (red) in HeLa (top), human foreskin fibroblasts (middle) and human lung fibroblasts (bottom).


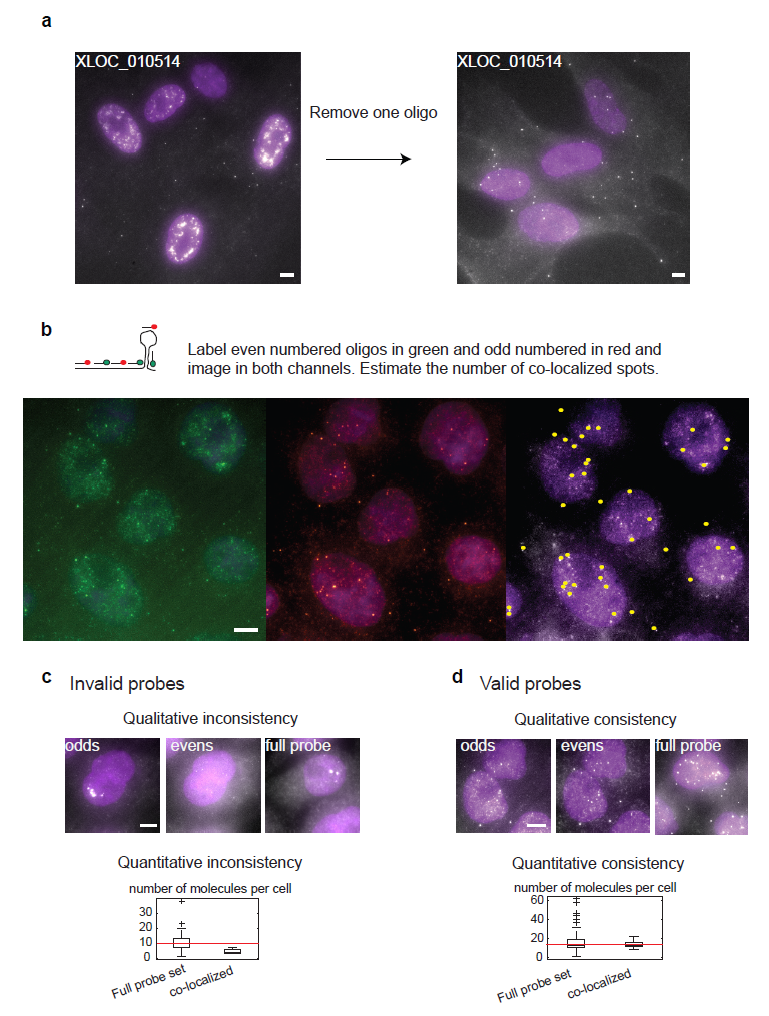


**Figure S2 | Two color co-localization approach for RNA-FISH probe set validation.**

**Figure S2 | Two color co-localization approach for RNA-FISH probe set validation**

**(a)** Florescence micrograph of the original probe set (left) targeting XLOC_010514 and the validated probe set after eliminating one oligonucleotide in hFF (right; **3.4** **Methods**). **(b)** Top: Illustration of the two color labeling of even and odd numbered oligonucleotides within a probe set for the co-localization probe validation assay. Bottom: Florescence micrographs of the NR_029435 probe set in HeLa of the even numbered oligonucleotides (left, green; Alexa 594), odd numbered oligonucleotides (middle, orange; Cy3) and co-localized spots over the even numbered set micrograph (right, yellow over white; Alexa 594). **(c)** Examples of qualitative and quantitative inconsistencies. Top: qualitative inconsistency between the localization pattern of MIAT in HeLa cells as determined by odd numbered oligonucleotides (left), even numbered (middle) and full probe set (right). The pattern matches between odd and full set, but not evens. Bottom: quantitative inconsistency between the distribution of RNA molecule counts (Box plots) based on the full probe set (left) and based on the number of co-localized spots when imaging in two colors (right). Red bar: medians. Whiskers are is 1.5* the inner quartile range. **(d)** Examples of qualitative and quantitative consistencies. As in *c* but for the valid probe set for NR_029435 in HeLa cells, which is both qualitatively (top) and quantitatively (bottom) consistent.


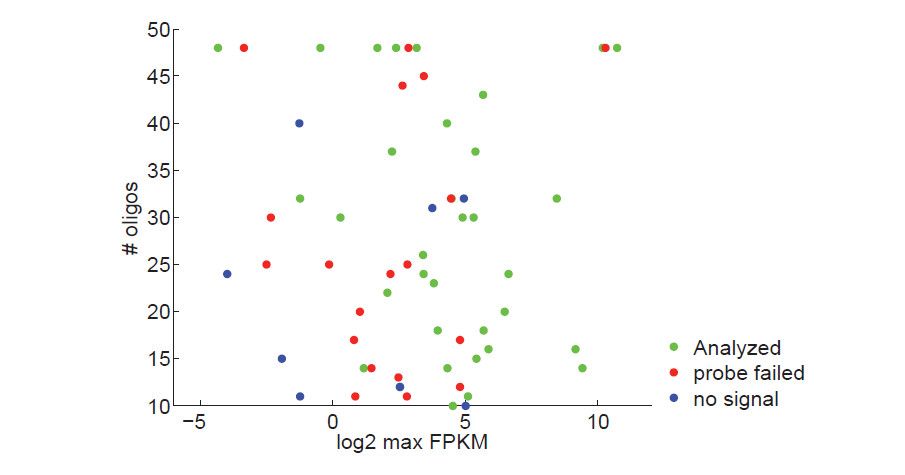


**Figure S3 | Dependency of probe set success on the number of oligonucleotides and lncRNA expression**

Scatter plot showing the relationship between the population average expression level as measured by RNA-Seq (X-axis, maximal log_2_(FPKM) across HeLa/hLF/hFF) and the number of oligonucleotides designed to target each lncRNA (Y-axis). Data points are colored by their classification to valid probe set included in the analysis (green), probe failed in the co-localization assay (red) or hybridization resulted with no signal (blue).

**
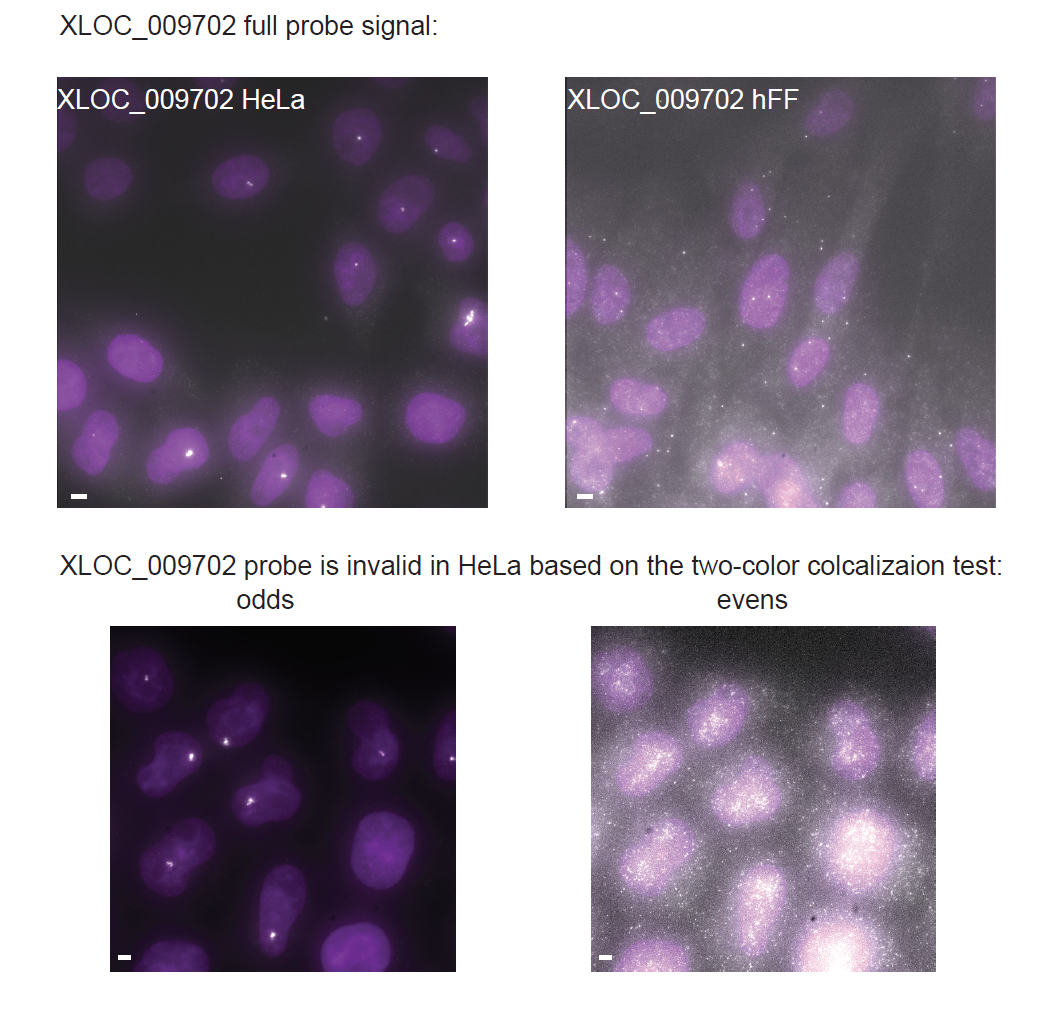
**

**Figure S4 | Qualitative inconsistency of the XLOC_009702 probe set in one cell type but not another**

Shown are micrographs for XLOC_009702 imaged with the full probe set (top) in either HeLa (top left) or hFF (top right), or with the odds and even probe sets in HeLa (bottom left and right, respectively). The probe set is valid in hFF but fails the two-color co-localization assay in HeLa cells.

**
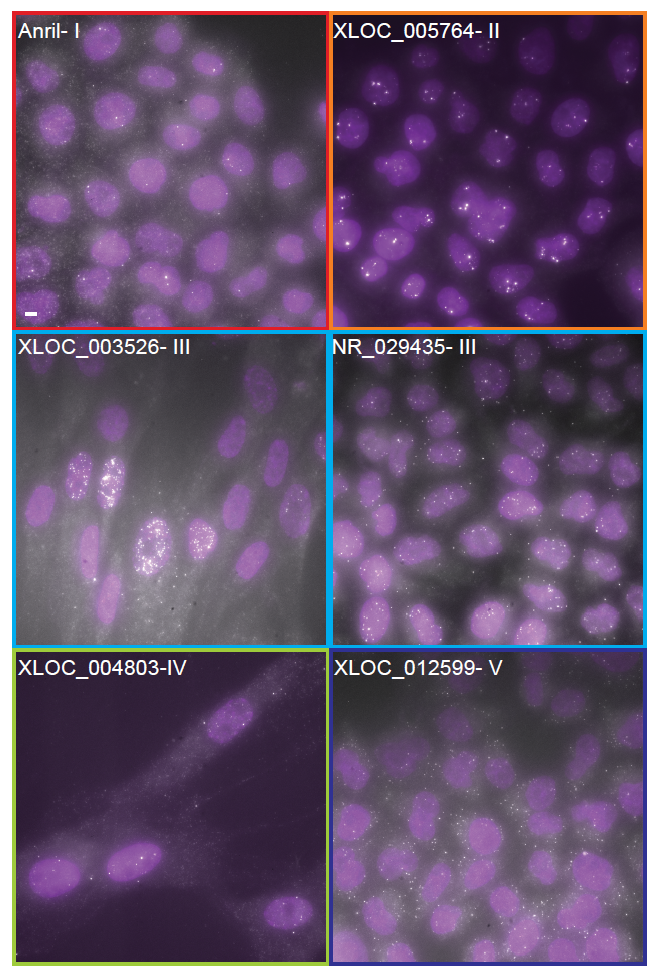
**

**Figure S5 | Cellular localization patterns**

Shown are representative whole image fields for selected examples from each pattern type I-V, as defined in *Figure 2*. Scale bar, 5 µm.

**
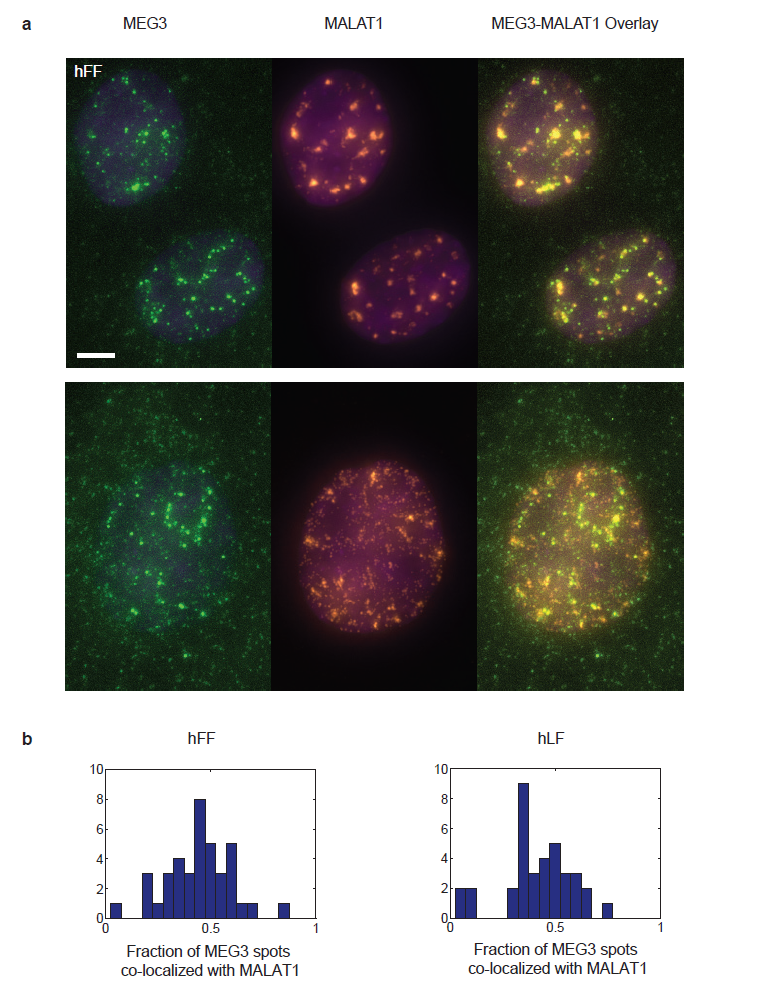
**

**Figure S6 | Co-localization of MEG3 and MALAT1**

**Figure S6 | Co-localization of MEG3 and MALAT1**

**(a)** Florescence micrograph of representative hFF nuclei expressing MEG3(left, green) and MALAT1(middle, red). Co-localization of MEG3 molecules and MALAT1 foci can be observed in the overlay images on the right. Top panel represent cases of high overlap (44% and 59% of MEG3 spots co-localize in the top and bottom nuclei respectively), where the bottom panel represents a case of low overlap (less than 6% of MEG3 spots overlap). Scale bar, 5 µm. **(b)** The distribution of the fraction of MEG3 spots that co-localize with MALAT1 in hFF (n=40, left) and hLF (n=37, right) cells.

**
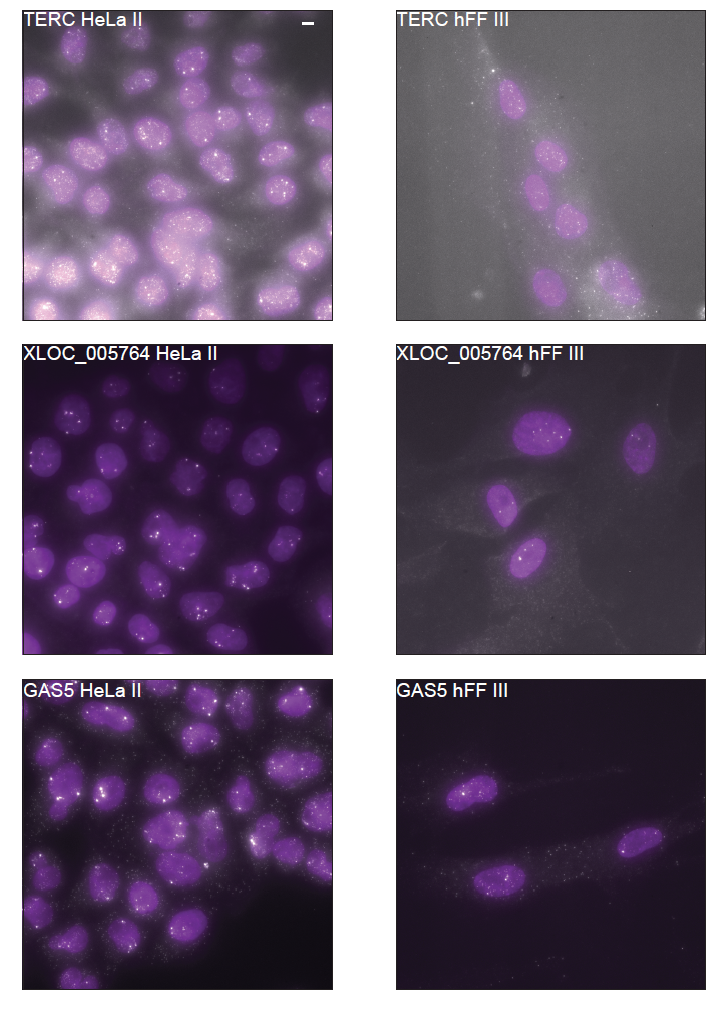
**

**Figure S7 | Presence of nuclear foci for the same lincRNA in one cell type but not another**

Florescence micrographs demonstrating a pattern switch for TERC (top), XLOC_005764 (middle) and GAS5 (bottom) between HeLa cells (left), where expression is higher and nuclear foci are identified, and hFF (right), where foci are absent. Scale bar, 5 µm.

**
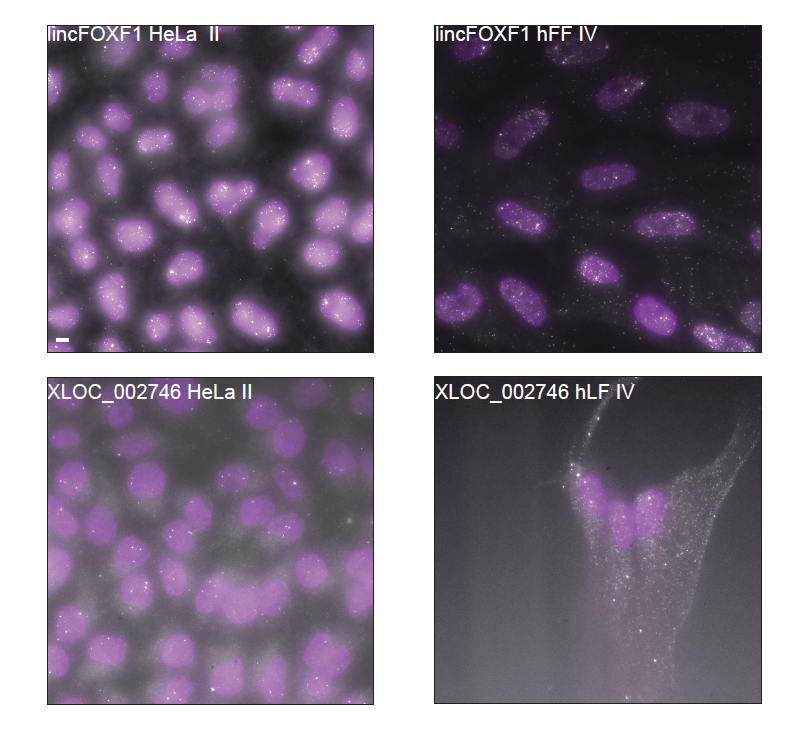
**

**Figure S8 | Presence of cytoplasmic transcripts for the same lincRNA in one cell type but not another**

Florescence micrograph demonstrating a pattern switch for lincFOXF1 (top) and XLOC_002746 (bottom) between fibroblasts (right), where expression is higher and cytoplasmic transcripts are identified, and HeLa cells (left) where only nuclear expression is detected. Scale bar, 5 µm.

**
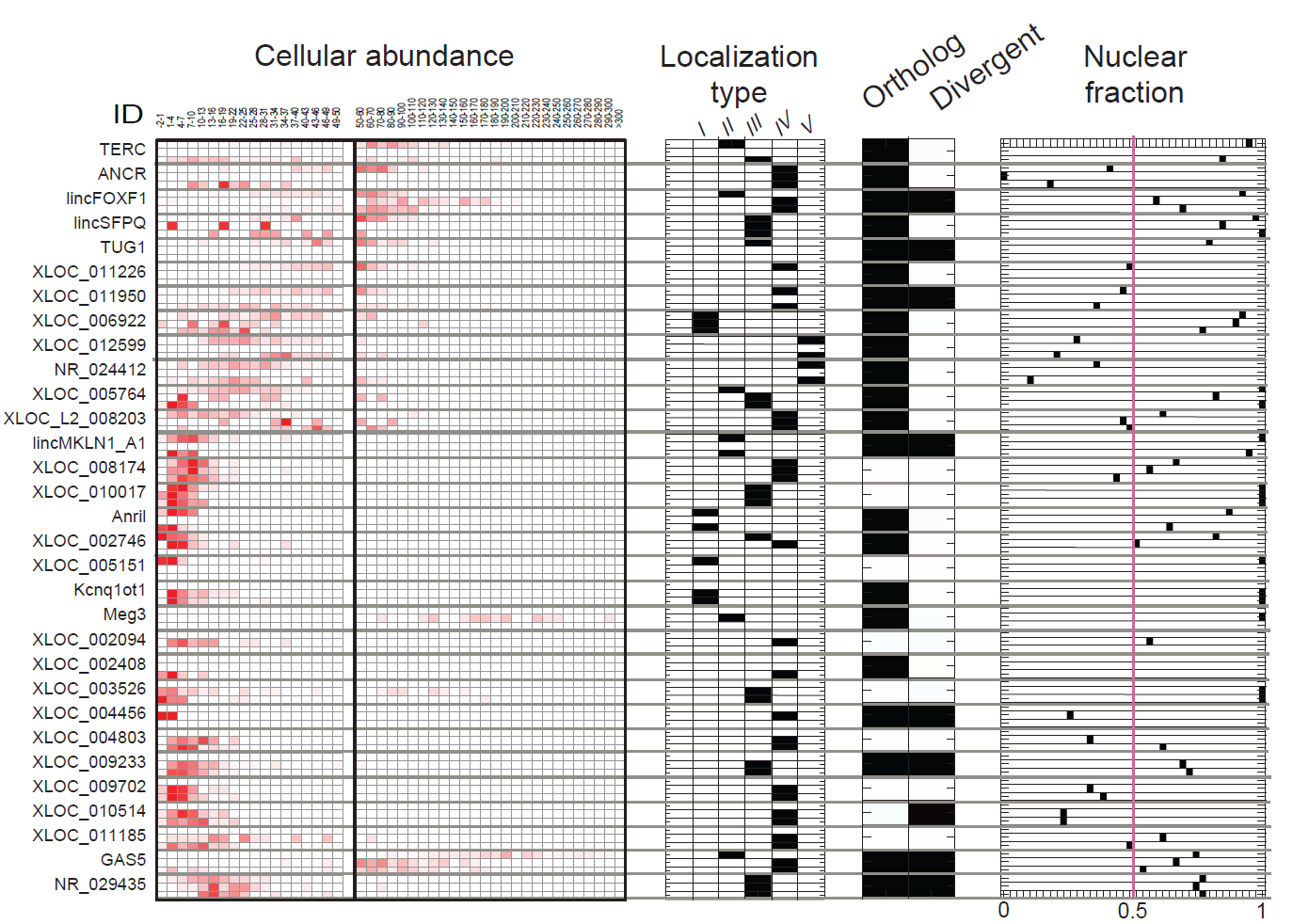
**

**Figure S9 | Summary of features for each lncRNA in the study**

For each lncRNA in the validated and quantitative probe set (rows) shown are from left to right: abundance in HeLa, hLF and hFF (top to bottom sub rows, respectively; presented as fractional density of single molecule counts as in *Figure 4a*); classification to I-V localization type as in *Figure 2*; having a mouse orthologs or being divergently transcribed; and the median fraction of spots localized to the nucleus in expressing cells. Cell types for which the probe set is not valid are blanked. Positive classifications are marked black in the three right most panels.

**
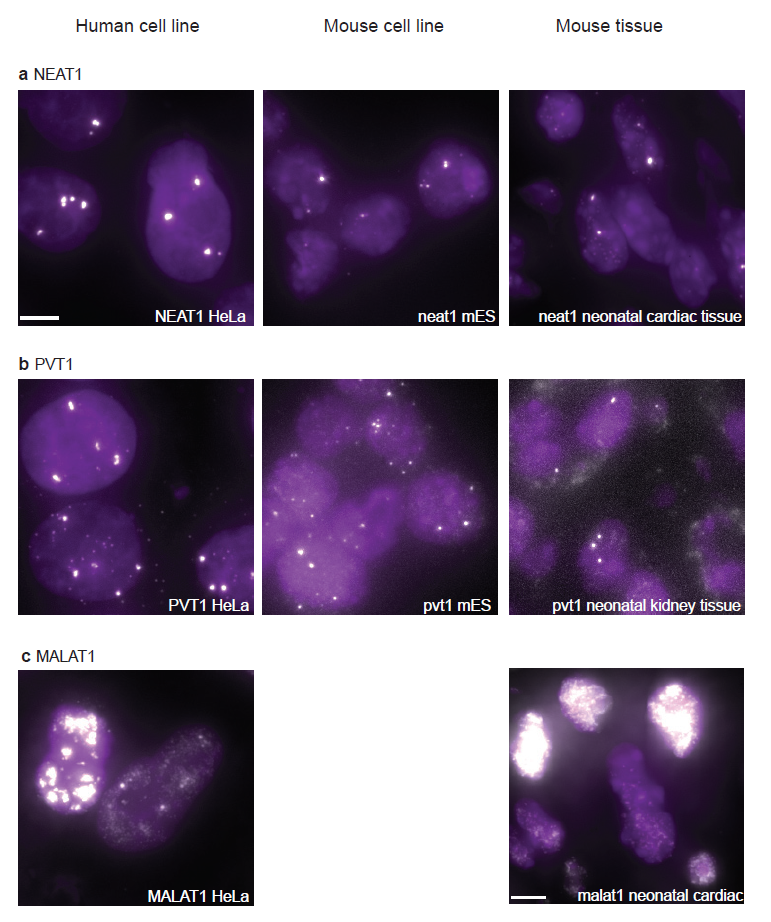
**

**Figure S10 | Consistent lncRNA localization patterns between cultures and tissue section and across tissue sections**

Florescence micrograph demonstrating consistent localization patterns of NEAT1 (a), PVT1 (b) and MALAT1 (c) across a human cell line (HeLa, left column), mouse line (mES, middle column) and mouse neonatal tissue (right column). Scale bar, 5 µm. All images refer to the top left scale bar, with the exception of the bottom right image.


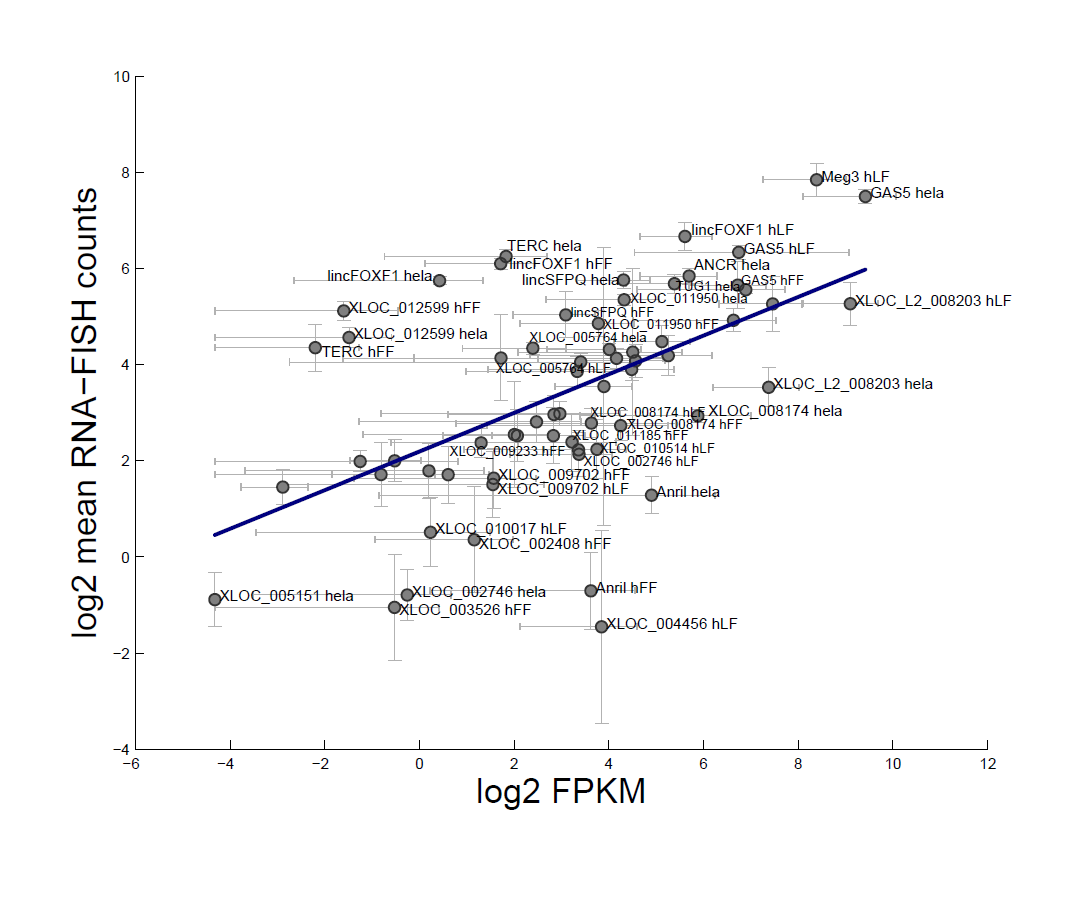


**Figure S11 | Correlation between mean (bulk) expression and single cell molecule count average**

Shown is a scatter plot comparing for each of the lncRNAs its expression level estimate in a bulk population from RNA-Seq (X-axis, log_2_(FPKM) from Cuffdiff2 [[6](#_ENREF_6)]) and the mean number of mRNA molecule identified by single molecule RNA-FISH (Y-axis, log_2_ scale). Error bars correspond to a 95% confidence interval. Regression line Y = 4.43X+2.1 (blue line; Pearson r=0.55). Off diagonal spots are labeled.


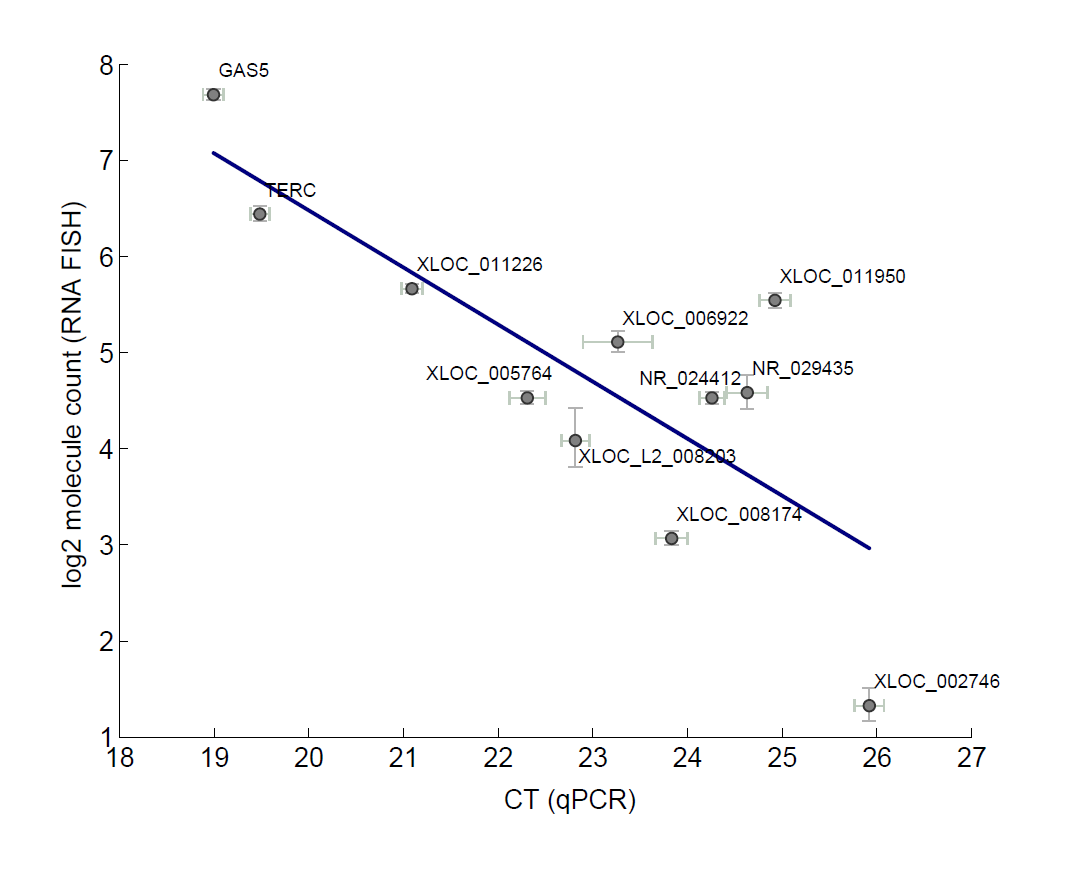


**Figure S12 | Correlation between qPCR bulk abundance estimates and single cell molecule count average**

Shown is a scatter plot comparing for each of the lncRNAs its expression level estimate in a bulk population from qPCR (represented by CT values, X- axis; the lower the CT the higher the expression) and the mean number of mRNA molecule identified by single molecule RNA-FISH (Y-axis, log_2_ scale). Error bars correspond to standard mean errors. qPCR CT values are averages of 3 biological replicates (2-4 technical replicates for each) normalized relative to GAPDH. Regression line Y = -0.59X+18.37 (blue line; Pearson r=-0.788).


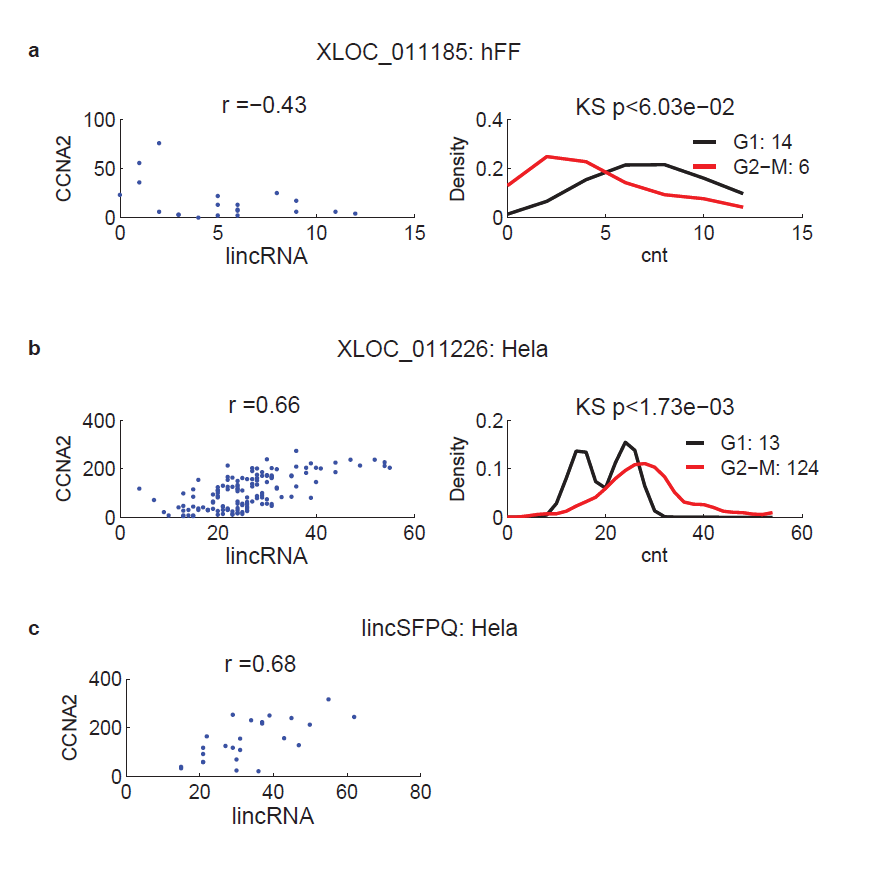


**Figure S13 | lncRNAs with cell-cycle correlated expression**

For each of three lncRNAs found to be significantly correlated with CCNA2, irrespective of cell volume, shown are a scatter plot (left) comparing the estimated molecule counts of the lncRNA (X-axis) and CCNA2 (Y-axis) in each (Pearson correlation r is noted on top) and histograms (right) of the molecule count distribution of the lncRNA in cells in G1 (black) or G2/S/M (red) (number of cells is in top right; P-value of testing whether the distributions differ using a Kolmogorov-Smirnov test is on top). Cells were classified as G1 or G2/S/M based on a threshold on CCNA2 levels as specified in the **3.4** **Methods**. (a) XLOC_011185 in hLF. (b) XLOC_011226 in HeLa. (c) lincSFPQ in HeLa. lincSFPQ did not have any cells in G1.


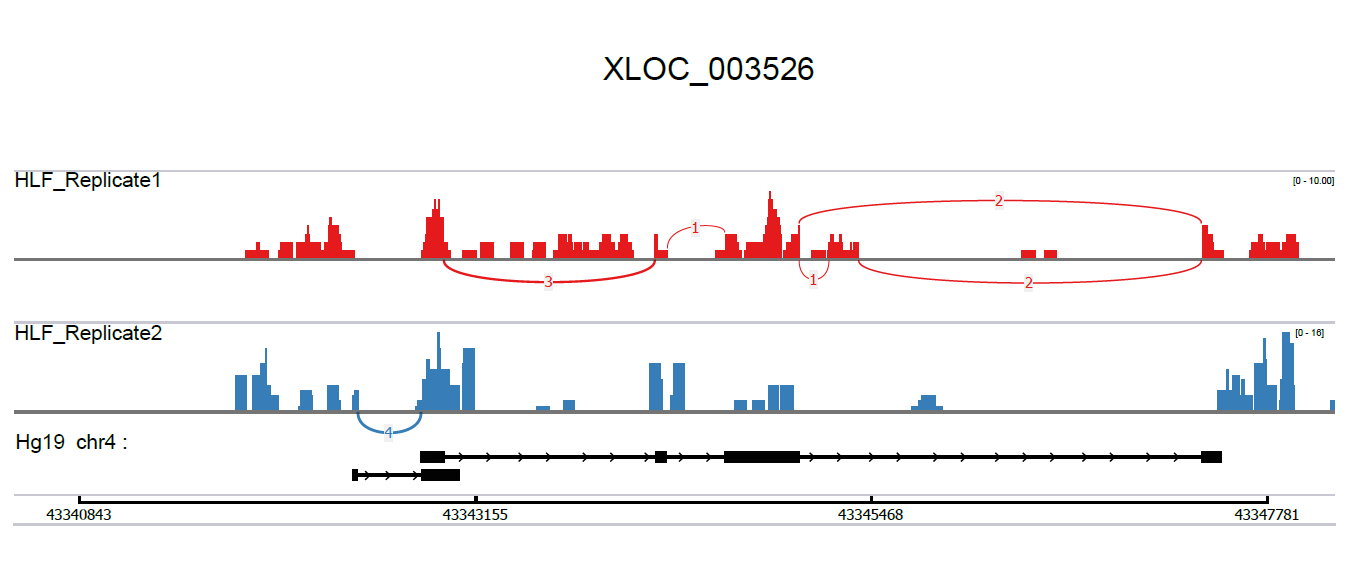


**Figure S14 | low RNA-Seq read coverage across the XLOC_003526 locus**

A Sashimi plot (as displayed by IGV [[10](#_ENREF_10)]), describing XLOC_003526 transcript reconstruction by Cufflinks [[11](#_ENREF_11)]. hLF RNA-Seq read alignment across the XLOC_003526 locus (top) from two replicates (red and blue coverage tracks) show the aggregate coverage of both spliced and un-spliced reads. Exon junctions spanned by the spliced reads are marked by bow curves. The number on top of the bow curve specifies how many spliced reads support this specific junction. There are only very few spliced reads in this data and these are all essential for reconstruction of the correct transcript (bottom).


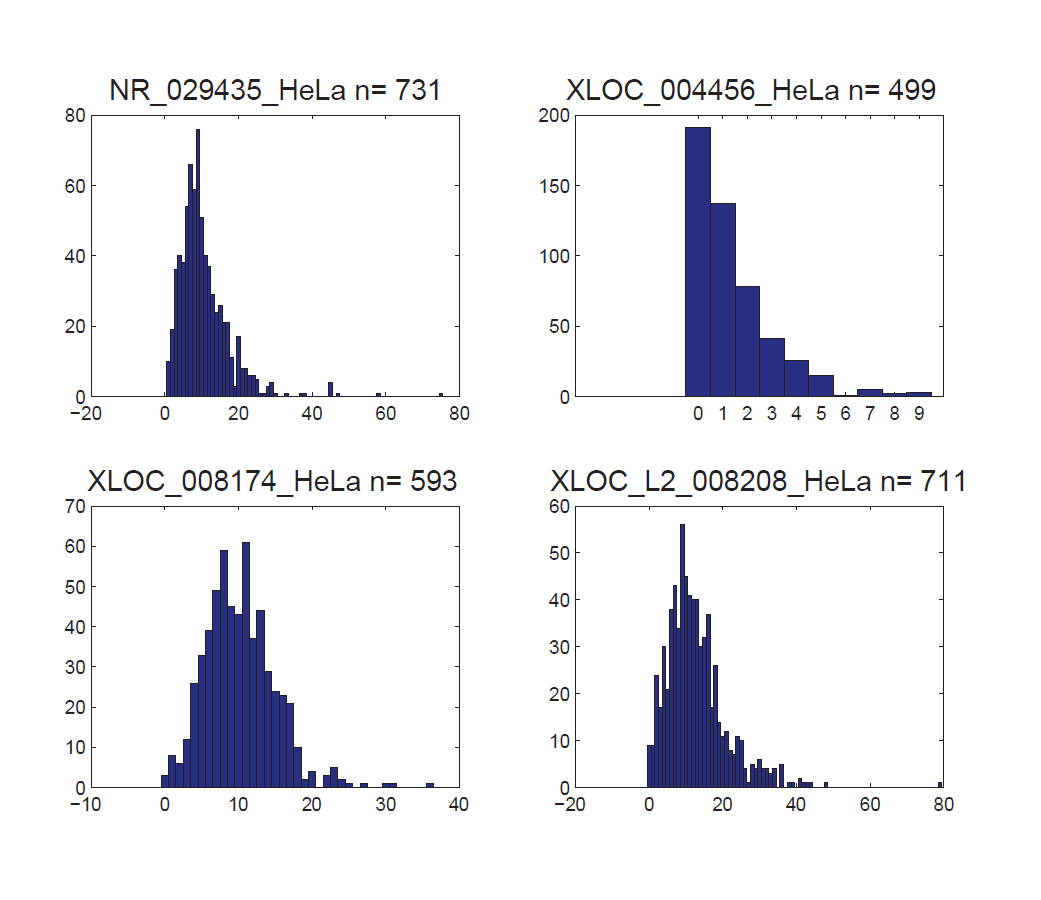


**Figure S15 | Transcript abundance distributions across large numbers of cells**

Shown are the distributions of number of molecules per cell detected when imaging many (n>498) cells for each of four different lncRNAs. The lncRNA ID, cell type and sample size (n) are specified on top.

**
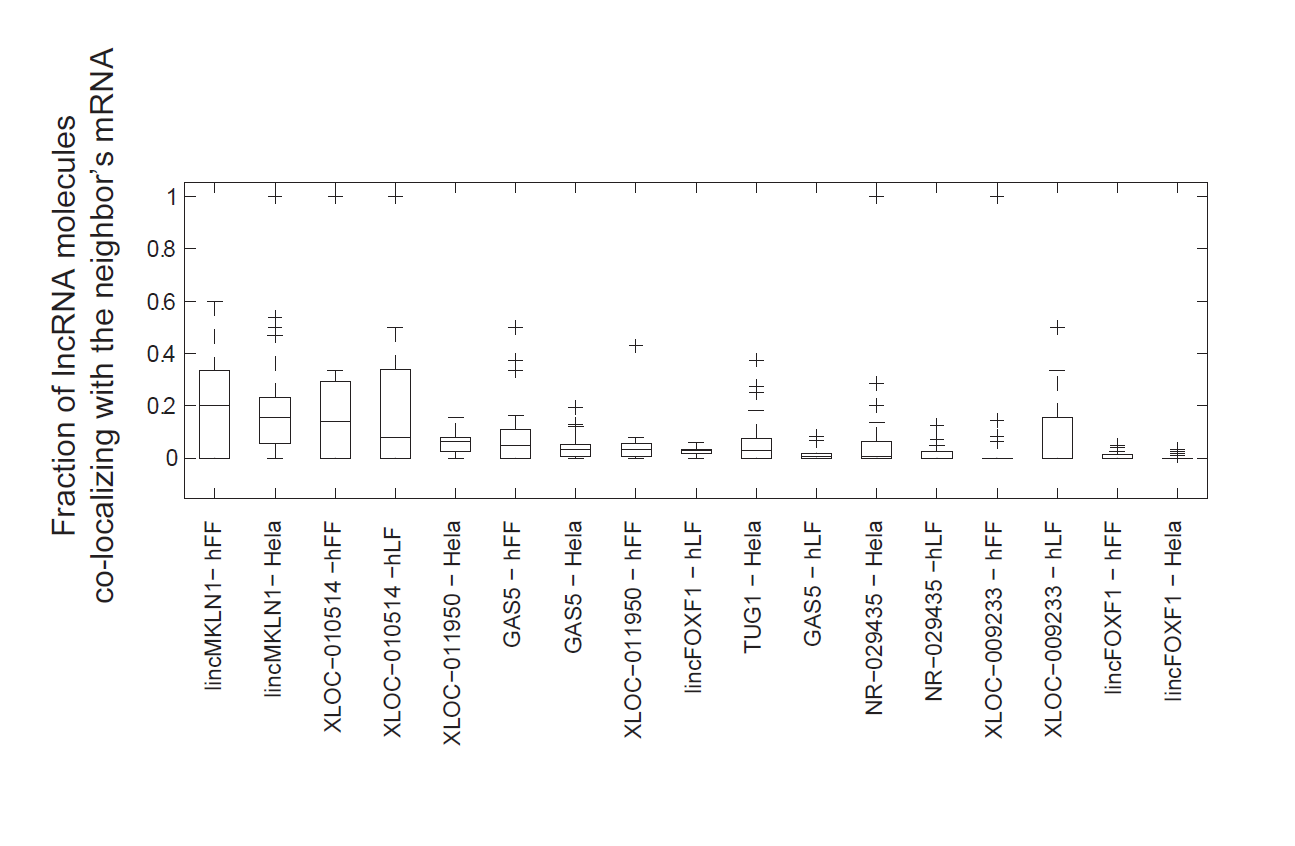
**

**Figure S16 | Divergently transcribed lncRNAs do not strongly co-localize with their neighboring mRNAs**

For each lncRNA-cell pair (X axis), shown is a Box plot of the distribution of the fraction of divergent lncRNA molecules that co-localize with their neighboring mRNA molecule across the imaged cells.

**
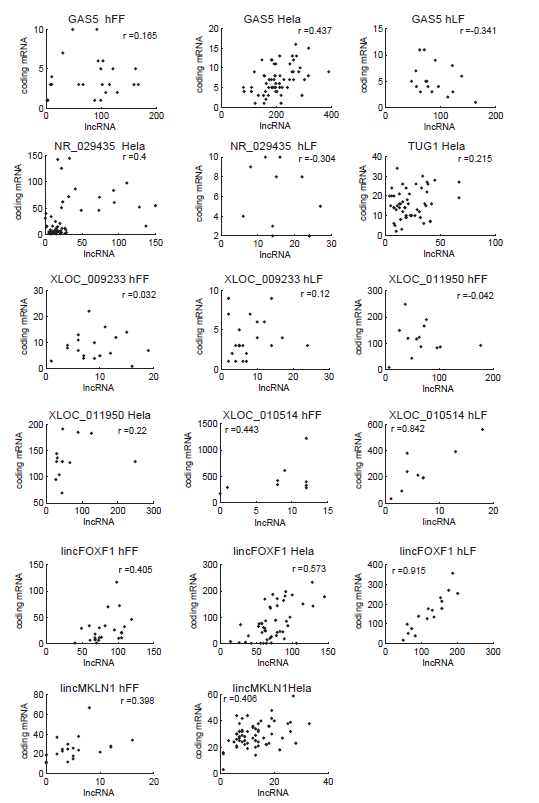
**

**Figure S17 | Correlation of expression between lncRNAs and their divergently transcribed mRNA neighbors**

For each lncRNA:mRNA:cell-type combination included in the analysis for divergent transcripts shown are scatter plots of the relation between the expression level in each cell of a lncRNA (X-axis, molecule count) and its cognate, divergently transcribed, mRNA (Y axis, molecule count). Pearson correlation coefficient (r) after removal of outliers (**Supplementary Methods**) is denoted on top.

**
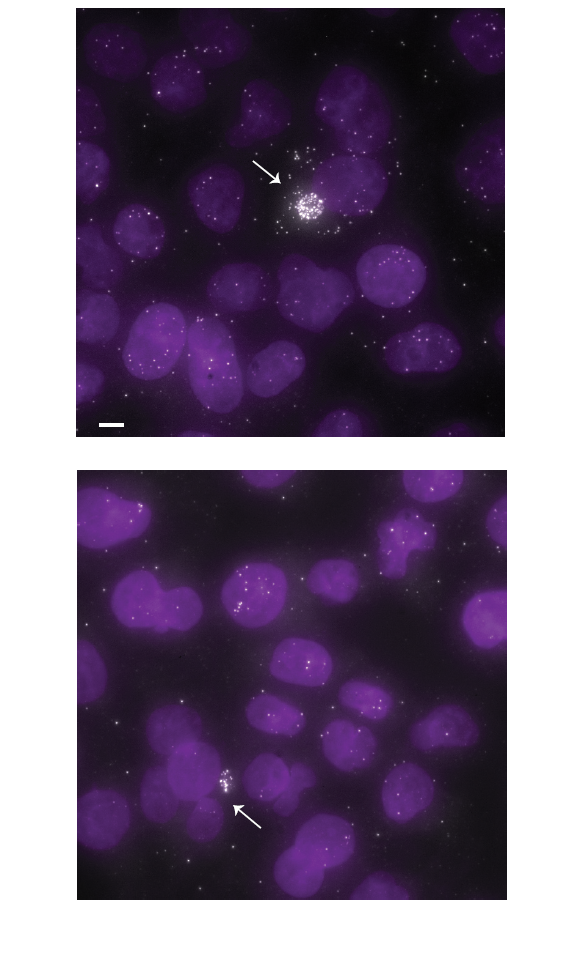
**

**Figure S18 | Two rare daughter HeLa cells express NR_029436 highly**

Shown are micrographs of NR_029436 with two rare, highly expressing, daughter HeLa cells (157 and 41 molecules per cell for the top and bottom images, respectively). Scale bar, 5 µm.

**
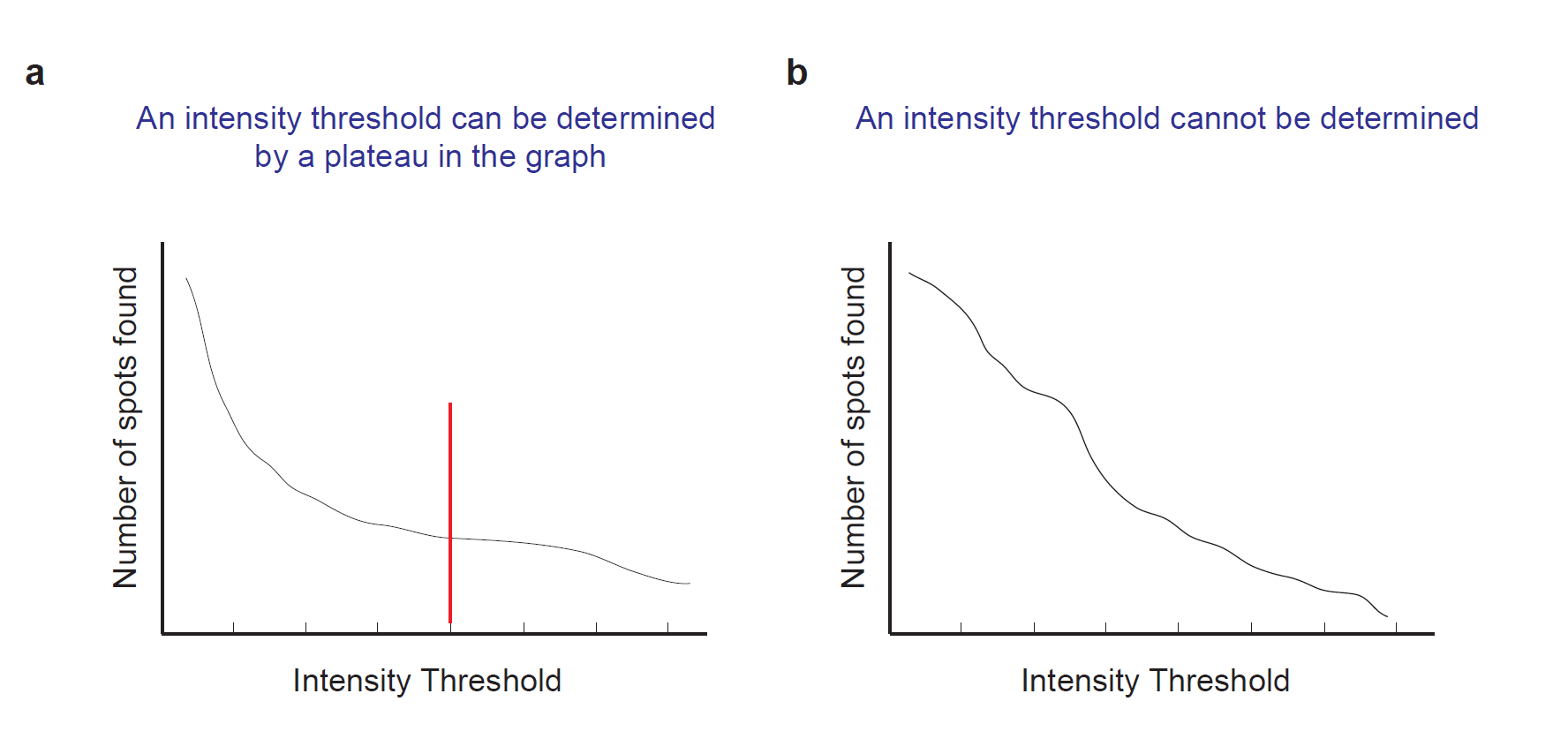
**

**Figure S19 | Determining a spot intensity threshold (X) to detect valid RNA spots**

Shown are illustration plots of the number of detected spots (Y axis) above each detection threshold (X-axis log2(intensity)) in **(a)** a case where a detection threshold can be determined by identifying a plateau in the graph; and **(b)** a case where a plateau is not identifiable. The latter is common for lncRNA images acquired with half of the probe set in the two-color co-localization assay.

**
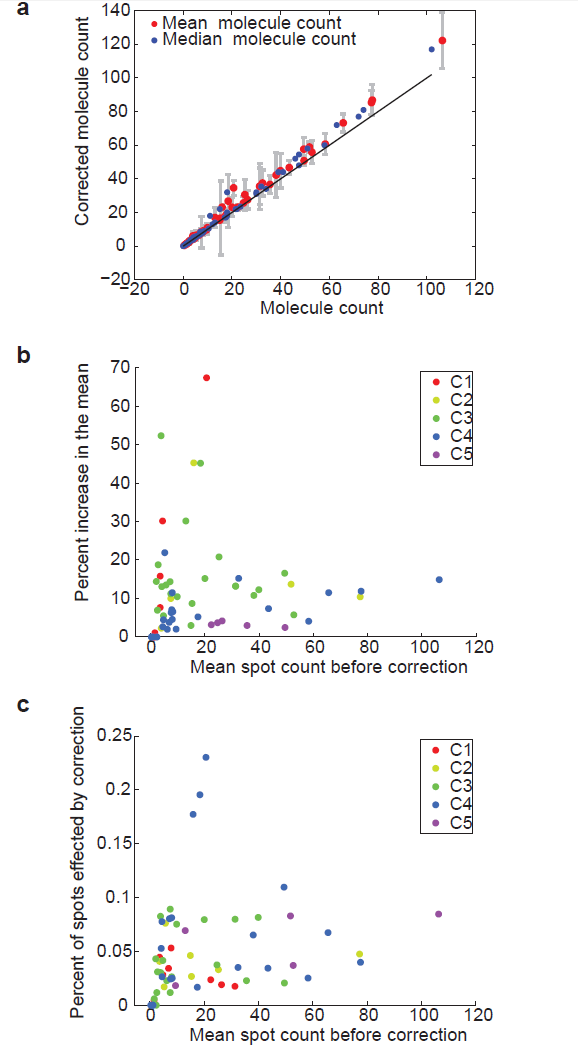
**

**Figure S20 | Single molecule count correction**

Scatter plot of the mean (red) and median (blue) total spot count before (X-axis) and after (Y-axis) spot count correction. Error bars (gray) are the standard error of the mean. Black line Y=X. **(b-c)** Scatter plots of the mean spot count before correction (X axis) *vs*. either the percent increase in the mean after applying the correction (*b*) or the mean percent of spots that were affected by the correction across the cells (*c*). Data points are colored by classification to I-V class types, as in *Figure 2 (*noted here as C1-C5, respectively*)*. Top 10% (n=6) highest data points exhibit similar behavior and are excluded from this figure for resolution purposes. Each data point describes the population of cells expressing a specific lncRNA in a specific cell type.

**Figure S21 | Probe set validation by two color co-localization. See following pages.**

Each row present three panels describing the same lncRNA in the three cell types assayed in this study. Each panel compares for that lincRNA:cell type pair, the count distribution as estimated by the full probe (full, s1) or by the spots that co-localize in the two-color assay (Co-loc, s2) by their: descriptive statistics (left), Box plots (middle), and quantile-quantile (QQ) plots (right). The descriptive statistics report from top to bottom: P-value of a Mann Whitney U test when comparing the two count distributions; r_o_=U/s1*s2, where U is the ranksum statistic, and s_i_ is the sample size in each set, provides an estimate from 0 to 1 of the extent of overlap between the two distributions, where r_o_=0.5 represents perfect similarity; sample set sizes for s1 and s2; and E, the log_2_FPKM expression estimate from RNA-Seq. Some panels are marked by a colored box on the top left with the following color code: yellow- quantitatively or qualitatively inconsistent signal (classification 2.1-2.3); purple – insufficient data due to technical reasons (classification 2.4-2.7); orange – no signal in either the full probe set or the two-color assay (classification 3); red - valid after manual recovery. A borderline case with a pattern that is similar to a valid pattern of the gene in another cell type (classification 1.5). Panels where a box is absent are valid sets. Classifications are specified in **Additional file: 3.1**.


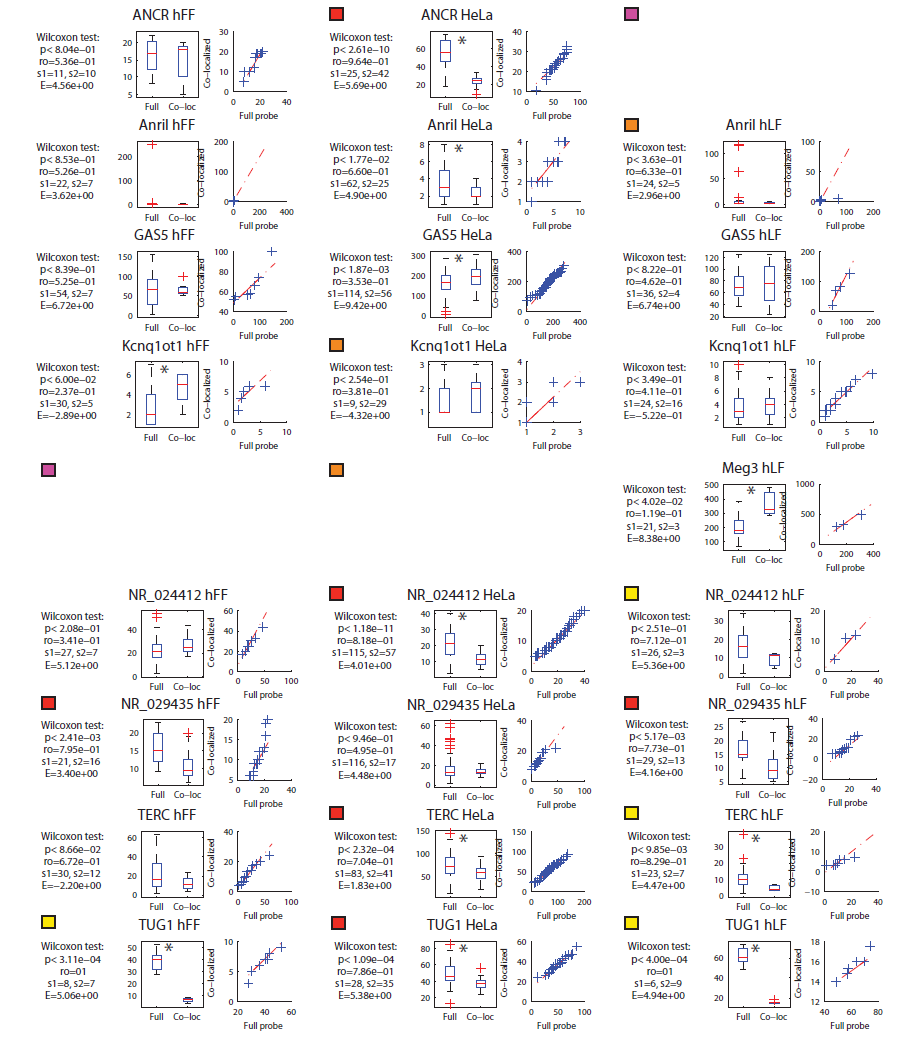


**Figure S21a | Probe set validation by two color co-localization. See legend above.**


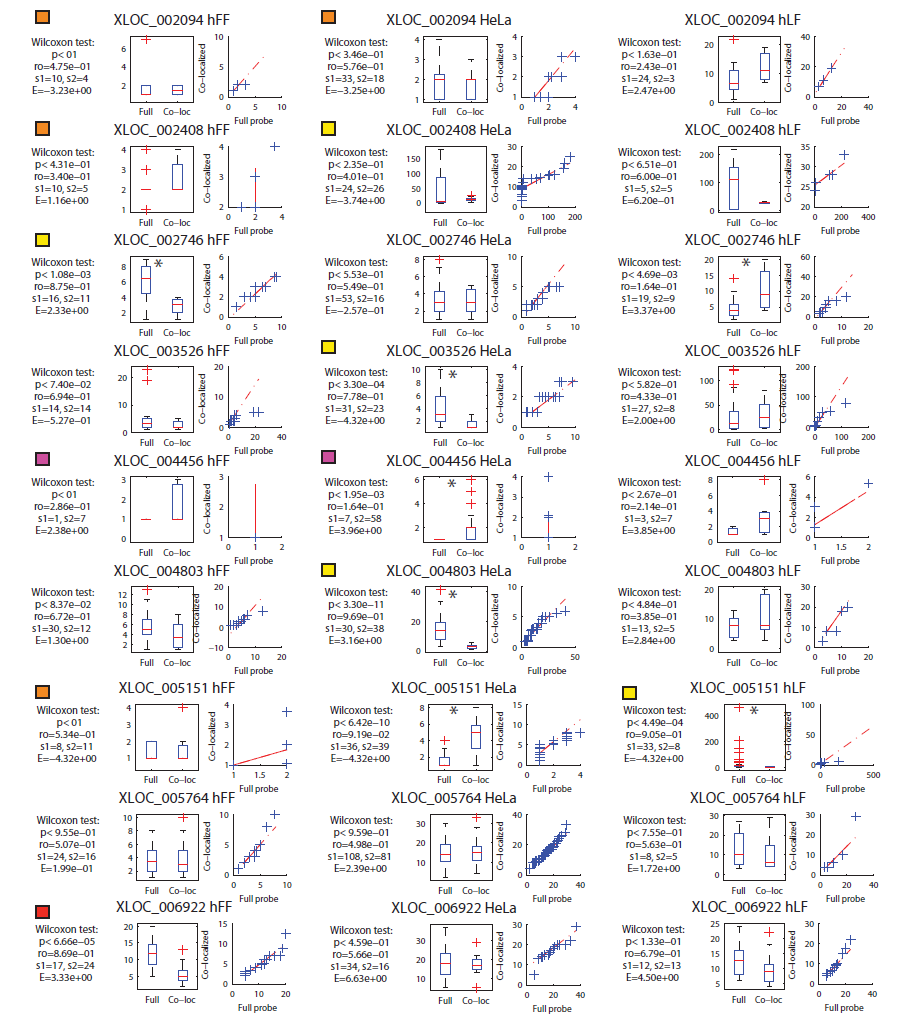


**Figure S21b | Probe set validation by two color co-localization. See legend above.**


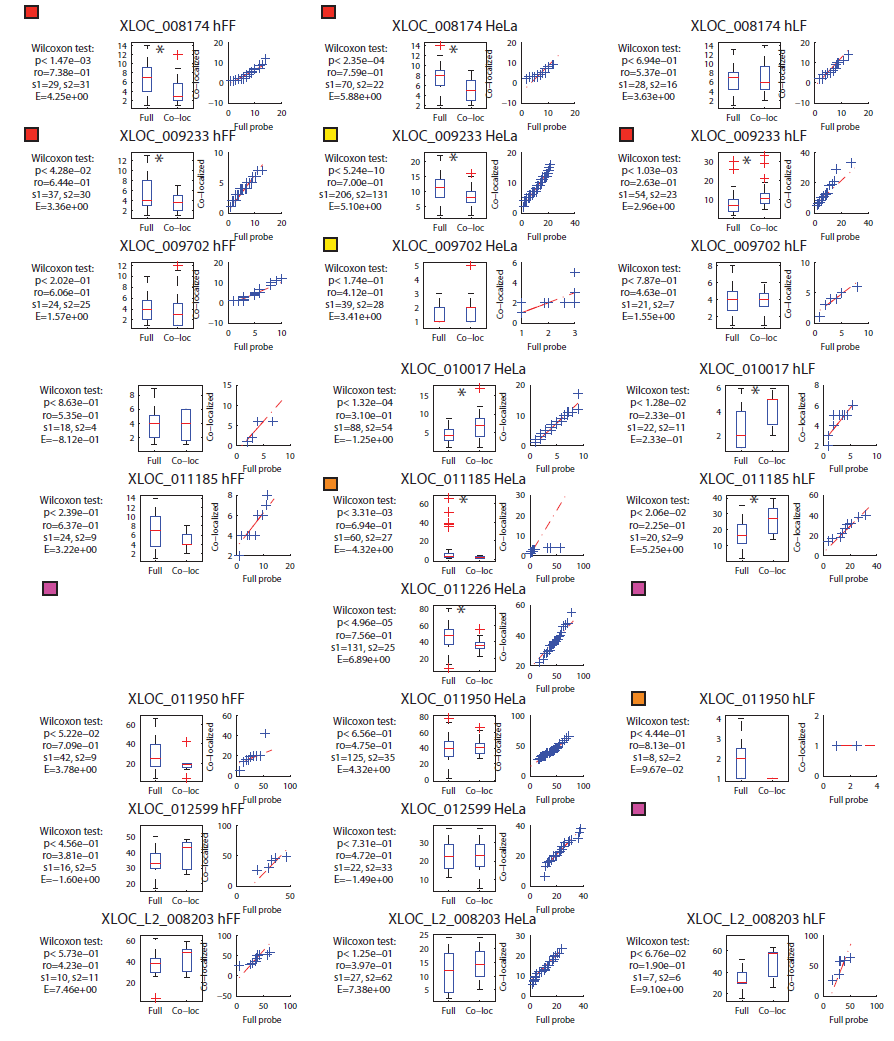


**Figure S21c | Probe set validation by two color co-localization. See legend above.**


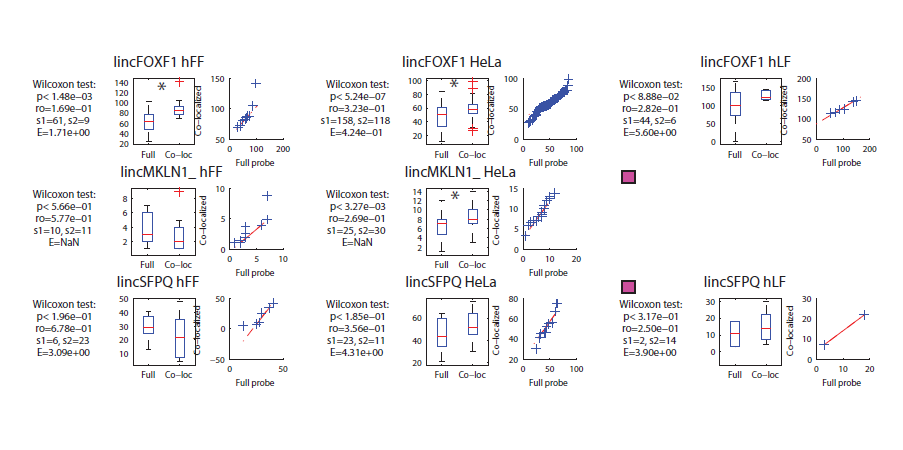


**Figure S21d | Probe set validation by two color co-localization. See legend above.**


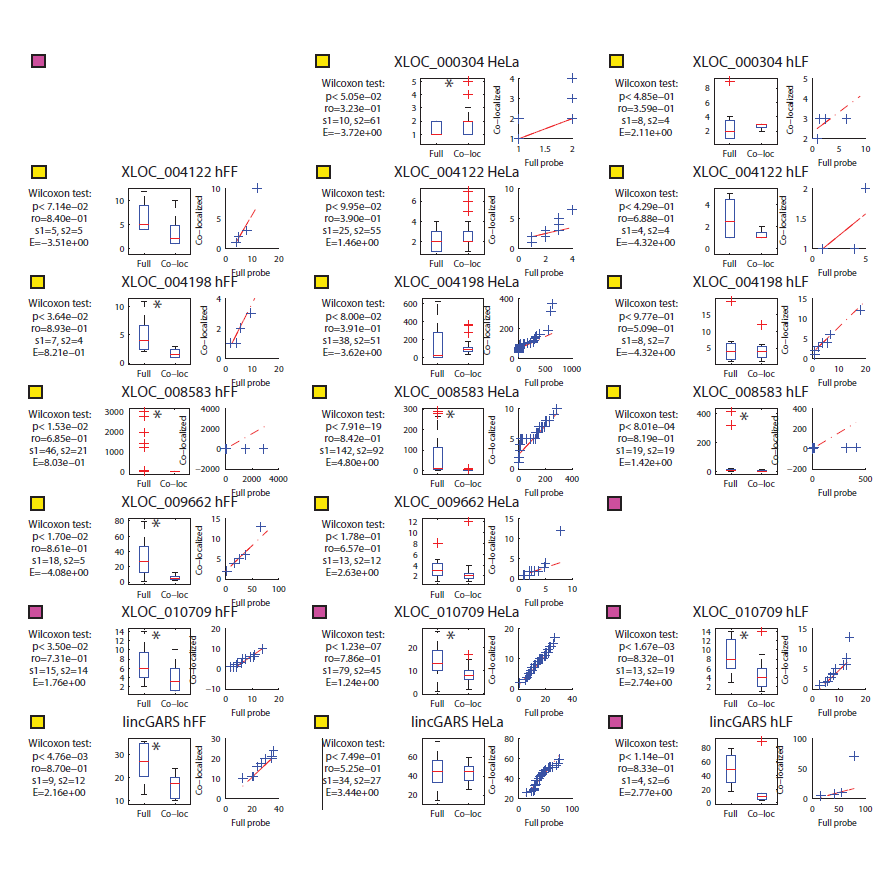


**Figure S21e | Probe set validation by two color co-localization. See legend above.**

**
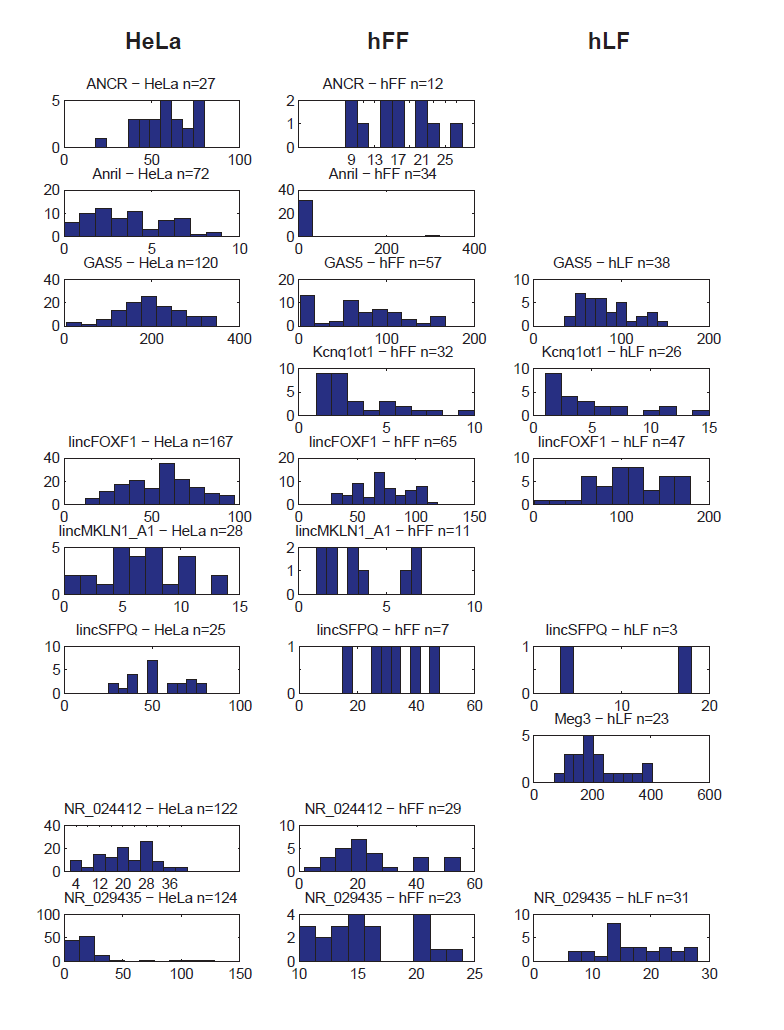
**

**Figure S22a | Molecule count distributions**

For each lincRNA (row) and each cell type (column) from the validated lincRNA:cell type set shown is a distribution of the molecule counts . The lncRNA and cell type as well as the total number of cells are noted on top of each distribution.


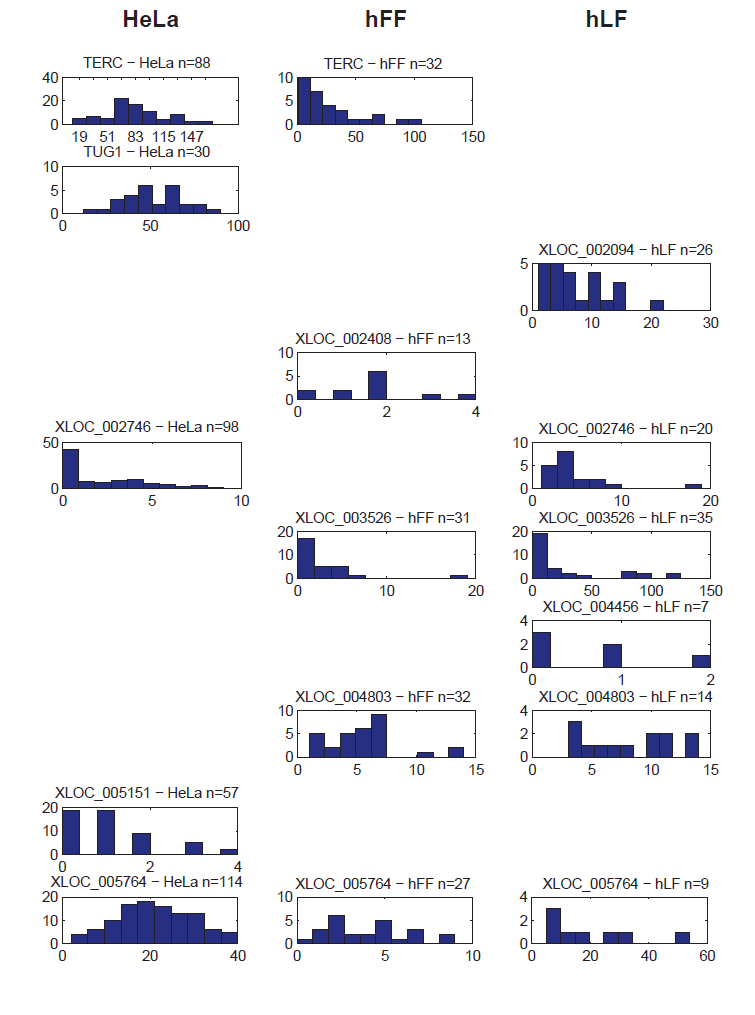


**Figure S22b | Molecule count distributions**

For each lincRNA (row) and each cell type (column) from the validated lincRNA:cell type set shown is a distribution of the molecule counts . The lncRNA and cell type as well as the total number of cells are noted on top of each distribution.


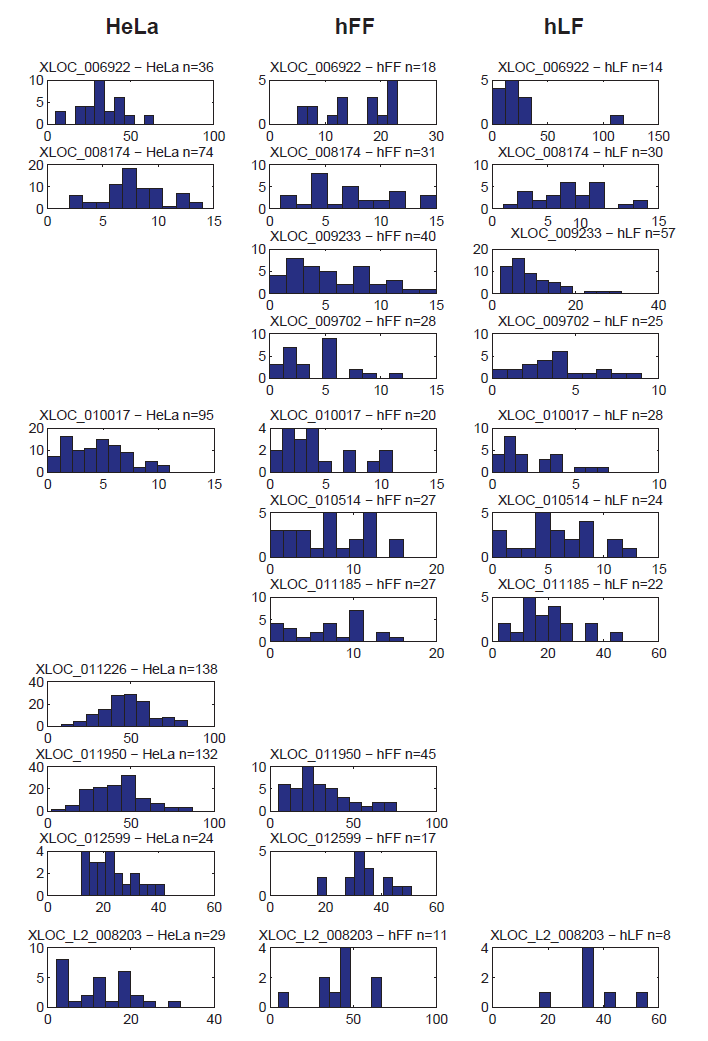


**Figure S22c | Molecule count distributions**

For each lincRNA (row) and each cell type (column) from the validated lincRNA:cell type set shown is a distribution of the molecule counts . The lncRNA and cell type as well as the total number of cells are noted on top of each distribution.

**Table S1| lncRNA-FISH candidate set characteristics.** “ValidSignal?” Indices : 1-yes 2-failed co-loc 3-nosignal.

| \| **Screen ID** \| **Valid**  **Signal?** \| **#Oligos** \| **Ortholog** \| **Divergent** \| **Number**  **Of**  **Exons** \| **Transcript**  **Len (bp)** \| **Tissue**  **Specificity** \| \| --- \| --- \| --- \| --- \| --- \| --- \| --- \| --- \| \| ANCR \| 1 \| 18 \| 1 \| 0 \| 3 \| 855 \| 0.087 \| \| ANRIL \| 1 \| 30 \| 1 \| 0 \| 19 \| 3834 \| 0.098 \| \| GAS5 \| 1 \| 14 \| 1 \| 1 \| 12 \| 632 \| 0.097 \| \| h19 \| 2 \| 32 \| 1 \| 0 \| 5 \| 2308 \| 0.129 \| \| KCNQ1OT1 \| 1 \| 48 \| 1 \| 0 \| 1 \| 91671 \| 0.175 \| \| lincFOXF1 \| 1 \| 43 \| 1 \| 1 \| 6 \| 6662 \| 0.195 \| \| lincGARS \| 2 \| 45 \| 0 \| 1 \| 7 \| 3871 \| 0.087 \| \| lincMKLN1 \| 1 \| 37 \| 1 \| 1 \| 4 \| 2948 \| 0.107 \| \| lincSFPQ \| 1 \| 40 \| 1 \| 0 \| 3 \| 2670 \| 0.087 \| \| MALAT1 \| 1 \| 48 \| 1 \| 0 \| 1 \| 8707 \| 0.086 \| \| MEG3 \| 1 \| 32 \| 1 \| 0 \| 8 \| 1722 \| 0.162 \| \| MIAT \| 2 \| 48 \| 1 \| 0 \| 4 \| 9982 \| 0.105 \| \| NEAT1 \| 1 \| 48 \| 1 \| 0 \| 1 \| 3735 \| 0.088 \| \| NR_024412 \| 1 \| 15 \| 1 \| 0 \| 3 \| 1127 \| 0.089 \| \| NR_029435 \| 1 \| 32 \| 1 \| 1 \| 4 \| 2442 \| 0.079 \| \| NRON \| 2 \| 48 \| 1 \| 0 \| 1 \| 2730 \| 0.287 \| \| TERC \| 1 \| 10 \| 1 \| 0 \| 1 \| 451 \| 0.236 \| \| TUG1 \| 1 \| 37 \| 1 \| 1 \| 3 \| 7104 \| 0.079 \| \| XIST \| 1 \| 48 \| 1 \| 0 \| 6 \| 19553 \| 0.179 \| \| XLOC_000304 \| 2 \| 24 \| 1 \| 1 \| 4 \| 952 \| 0.098 \| \| XLOC_002094 \| 1 \| 12 \| 0 \| 0 \| 4 \| 717 \| 0.834 \| \| XLOC_002263 \| 2 \| 25 \| 1 \| 0 \| 8 \| 1619 \| 0.096 \| \| XLOC_002408 \| 1 \| 14 \| 1 \| 0 \| 3 \| 1058 \| 0.131 \| \| XLOC_002746 \| 1 \| 24 \| 1 \| 0 \| 7 \| 9656 \| 0.112 \| \| XLOC_003526 \| 1 \| 22 \| 0 \| 0 \| 5 \| 990 \| 0.417 \| \| XLOC_004122 \| 2 \| 14 \| 1 \| 1 \| 3 \| 1194 \| 0.166 \| \| XLOC_004198 \| 2 \| 11 \| 0 \| 0 \| 4 \| 1771 \| 0.394 \| \| XLOC_004456 \| 1 \| 18 \| 1 \| 1 \| 4 \| 730 \| 0.091 \| \| XLOC_004803 \| 1 \| 48 \| 0 \| 0 \| 2 \| 3120 \| 0.133 \| \| XLOC_005151 \| 1 \| 48 \| 0 \| 0 \| 3 \| 3047 \| 0.654 \| \| XLOC_005764 \| 1 \| 48 \| 1 \| 0 \| 4 \| 2933 \| 0.155 \| \| XLOC_006198 \| 2 \| 17 \| 0 \| 0 \| 5 \| 1091 \| 0.317 \| \| XLOC_006922 \| 1 \| 24 \| 1 \| 0 \| 3 \| 1218 \| 0.131 \| \| XLOC_008174 \| 1 \| 16 \| 0 \| 0 \| 4 \| 2886 \| 0.083 \| \| XLOC_008583 \| 2 \| 12 \| 0 \| 0 \| 3 \| 988 \| 0.102 \| \| XLOC_009233 \| 1 \| 11 \| 1 \| 1 \| 3 \| 673 \| 0.091 \| \| XLOC_009447 \| 3 \| 24 \| 1 \| 0 \| 2 \| 3206 \| 0.234 \| \| XLOC_009474 \| 2 \| 48 \| 1 \| 0 \| 2 \| 3710 \| 0.087 \| \| XLOC_009662 \| 2 \| 44 \| 0 \| 0 \| 3 \| 2716 \| 0.443 \| \| XLOC_009702 \| 1 \| 26 \| 0 \| 0 \| 5 \| 1895 \| 0.228 \| \| XLOC_010017 \| 1 \| 30 \| 0 \| 0 \| 7 \| 2207 \| 0.135 \| \| XLOC_010202 \| 2 \| 17 \| 1 \| 1 \| 2 \| 1454 \| 0.084 \| \| XLOC_010263 \| 3 \| 32 \| 1 \| 1 \| 3 \| 6207 \| 0.172 \| \| XLOC_010514 \| 1 \| 23 \| 0 \| 1 \| 2 \| 1985 \| 0.162 \| \| XLOC_010556 \| 2 \| 25 \| 1 \| 0 \| 5 \| 1589 \| 0.224 \| \| XLOC_010709 \| 2 \| 11 \| 0 \| 1 \| 2 \| 1083 \| 0.133 \| \| XLOC_010853 \| 3 \| 40 \| 1 \| 0 \| 4 \| 863 \| 0.360 \| \| XLOC_011185 \| 1 \| 30 \| 0 \| 0 \| 3 \| 4496 \| 0.114 \| \| XLOC_011226 \| 1 \| 20 \| 1 \| 0 \| 5 \| 8974 \| 0.084 \| \| XLOC_011264 \| 2 \| 25 \| 0 \| 0 \| 3 \| 2516 \| 0.208 \| \| XLOC_011950 \| 1 \| 14 \| 1 \| 1 \| 5 \| 2768 \| 0.123 \| \| XLOC_012187 \| 2 \| 20 \| 1 \| 0 \| 2 \| 3690 \| 0.218 \| \| XLOC_012192 \| 2 \| 13 \| 1 \| 0 \| 4 \| 3169 \| 0.090 \| \| XLOC_012197 \| 3 \| 12 \| 0 \| 0 \| 4 \| 1304 \| 0.209 \| \| XLOC_012564 \| 3 \| 15 \| 1 \| 0 \| 5 \| 7748 \| 0.499 \| \| XLOC_012599 \| 1 \| 32 \| 1 \| 0 \| 5 \| 2455 \| 0.149 \| \| XLOC_012980 \| 3 \| 10 \| 0 \| 0 \| 2 \| 342 \| 0.083 \| \| XLOC_013841 \| 2 \| 30 \| 1 \| 1 \| 2 \| 2794 \| 0.234 \| \| XLOC_014160 \| 3 \| 11 \| 1 \| 0 \| 8 \| 1958 \| 0.353 \| \| XLOC_L2_008203 \| 1 \| 16 \| 1 \| 0 \| 2 \| 793 \| 0.099 \| \| XLOC_L2_010926 \| 3 \| 31 \| 1 \| 0 \| 3 \| 2603 \| 0.083 \| |  |  |  |  |  |  |  |  |  |  |  |
| --- | --- | --- | --- | --- | --- | --- | --- | --- | --- | --- | --- | --- | --- | --- | --- | --- | --- | --- | --- | --- | --- | --- | --- | --- | --- | --- | --- | --- | --- | --- | --- | --- | --- | --- | --- | --- | --- | --- | --- | --- | --- | --- | --- | --- | --- | --- | --- | --- | --- | --- | --- | --- | --- | --- | --- | --- | --- | --- | --- | --- | --- | --- | --- | --- | --- | --- | --- | --- | --- | --- | --- | --- | --- | --- | --- | --- | --- | --- | --- | --- | --- | --- | --- | --- | --- | --- | --- | --- | --- | --- | --- | --- | --- | --- | --- | --- | --- | --- | --- | --- | --- | --- | --- | --- | --- | --- | --- | --- | --- | --- | --- | --- | --- | --- | --- | --- | --- | --- | --- | --- | --- | --- | --- | --- | --- | --- | --- | --- | --- | --- | --- | --- | --- | --- | --- | --- | --- | --- | --- | --- | --- | --- | --- | --- | --- | --- | --- | --- | --- | --- | --- | --- | --- | --- | --- | --- | --- | --- | --- | --- | --- | --- | --- | --- | --- | --- | --- | --- | --- | --- | --- | --- | --- | --- | --- | --- | --- | --- | --- | --- | --- | --- | --- | --- | --- | --- | --- | --- | --- | --- | --- | --- | --- | --- | --- | --- | --- | --- | --- | --- | --- | --- | --- | --- | --- | --- | --- | --- | --- | --- | --- | --- | --- | --- | --- | --- | --- | --- | --- | --- | --- | --- | --- | --- | --- | --- | --- | --- | --- | --- | --- | --- | --- | --- | --- | --- | --- | --- | --- | --- | --- | --- | --- | --- | --- | --- | --- | --- | --- | --- | --- | --- | --- | --- | --- | --- | --- | --- | --- | --- | --- | --- | --- | --- | --- | --- | --- | --- | --- | --- | --- | --- | --- | --- | --- | --- | --- | --- | --- | --- | --- | --- | --- | --- | --- | --- | --- | --- | --- | --- | --- | --- | --- | --- | --- | --- | --- | --- | --- | --- | --- | --- | --- | --- | --- | --- | --- | --- | --- | --- | --- | --- | --- | --- | --- | --- | --- | --- | --- | --- | --- | --- | --- | --- | --- | --- | --- | --- | --- | --- | --- | --- | --- | --- | --- | --- | --- | --- | --- | --- | --- | --- | --- | --- | --- | --- | --- | --- | --- | --- | --- | --- | --- | --- | --- | --- | --- | --- | --- | --- | --- | --- | --- | --- | --- | --- | --- | --- | --- | --- | --- | --- | --- | --- | --- | --- | --- | --- | --- | --- | --- | --- | --- | --- | --- | --- | --- | --- | --- | --- | --- | --- | --- | --- | --- | --- | --- | --- | --- | --- | --- | --- | --- | --- | --- | --- | --- | --- | --- | --- | --- | --- | --- | --- | --- | --- | --- | --- | --- | --- | --- | --- | --- | --- | --- | --- | --- | --- | --- | --- | --- | --- | --- | --- | --- | --- | --- | --- | --- | --- | --- | --- | --- | --- | --- | --- | --- | --- | --- | --- | --- | --- | --- | --- | --- | --- | --- | --- | --- | --- | --- | --- | --- | --- | --- | --- | --- | --- | --- | --- | --- | --- | --- | --- | --- | --- | --- | --- | --- | --- | --- | --- | --- | --- | --- | --- | --- | --- | --- | --- | --- | --- | --- | --- | --- | --- | --- | --- | --- | --- | --- | --- | --- | --- | --- | --- | --- |
|  |  |  | |  |  |  |  |  |  |  |  |
|  |  |  | |  |  |  |  |  |  |  |  |
|  |  |  | |  |  |  |  |  |  |  |  |
|  |  |  | |  |  |  |  |  |  |  |  |
|  |  |  | |  |  |  |  |  |  |  |  |
|  |  |  |  |  |  |  |  |  |  |  |  |
|  |  |  | |  |  |  |  |  |  |  |  |
|  |  |  | |  |  |  |  |  |  |  |  |
|  |  |  | |  |  |  |  |  |  |  |  |
|  |  |  | |  |  |  |  |  |  |  |  |
|  |  |  | |  |  |  |  |  |  |  |  |
|  |  |  | |  |  |  |  |  |  |  |  |
|  |  |  | |  |  |  |  |  |  |  |  |
|  |  |  | |  |  |  |  |  |  |  |  |
|  |  |  | |  |  |  |  |  |  |  |  |
|  |  |  | |  |  |  |  |  |  |  |  |
|  |  |  | |  |  |  |  |  |  |  |  |
|  |  |  | |  |  |  |  |  |  |  |  |
|  |  |  | |  |  |  |  |  |  |  |  |
|  |  |  | |  |  |  |  |  |  |  |  |
|  |  | |  |  |  |  |  |  |  |  |  |
|  |  |  | |  |  |  |  |  |  |  |  |
| **Table S2\| “classic” lncRNAs imaged in this study.** A previous reverence that included RNA-FISH of the corresponding gene, is specified if available.   \| **Name** \| **Previous publication with RNAFISH** \| **Pubmed ID** \| **Cell type in previous study** \| **Consistent?** \| \| --- \| --- \| --- \| --- \| --- \| \| DANCR \| NA \|  \|  \| NA \| \| ANRIL \| Yap et al. Mol Cell. 2010 \| PMID: 20541999 \| prostate cancer cell \| Signal in previous paper is not punctate. Single molecules cannot be defined \| \| GAS5 \| Kino et al. Sci. Signal. 2010 \| PMID: 20124551 \| HeLa \| yes \| \| KCNQ1OT1 \| Terranova et al. Mol. Cell 2008 \| PMID: 18848501 \| mouse trophectodermal stem (TS) cells \| yes \| \| lincFOXF1 \| Khalil et al. PNAS 2009 \| PMID: 19571010 \| hFF \| yes \| \| lincMKLN1 \| Khalil et al. PNAS 2009 \| PMID: 19571010 \| hFF \| yes \| \| lincSFPQ \| Khalil et al. PNAS 2009 \| PMID: 19571010 \| hFF \| yes \| \| MALAT1 \| Tripathi et al. Cell 2010 \| PMID: 20797886 \| HeLa \| yes \| \| MEG33 \| NA \|  \| \| NA \| \| MIAT \| Tsuiji et al. Genes toCells 2011 \| PMID: 21463453 \| DF1 HeLa and chicken spinal cord cells \| failed probe in our screen \| \| NEAT1 \| Clemson et al. Mol Cell 2009 \| PMID: 19217333 \| HeLa \| yes \| \| TERC \| Zhu et al. 2004 \| PMID: 14528011 \| HeLa HFF-Bjs \| Overall yes \| \| TUG1 \| Yang et al. cell 2011;Khalil et al. PNAS 2009 \| PMID: 22078878 ;PMID: 19571010 \| HeLa;hFF \| yes; yes \| \| XIST \| Hansen et al. PNAS 1998 ; Clemson et al. JCB 1996 ; Brown et al. Cell 1992 \| PMID: 9560241; PMID: 8636206; PMID: 1423611 \| normal human fibrblasts \| yes \| \| lincGARS \| NA \|  \|  \| no signal in our study \| \| NRON \| NA \|  \|  \| no signal in our study \| \| H19 \| Jouvenot et al. Curr. Bio. 2009 \| PMID: 10531031 \| mouse :E13.5 fetal liver \| no signal in our study \| |  |  | |  |  |  |  |  |  |  |  |
|  |  | |  |  |  |  |  |  |  |  |  |
|  |  | |  |  |  |  |  |  |  |  |  |
|  |  | |  |  |  |  |  |  |  |  |  |
|  |  | |  |  |  |  |  |  |  |  |  |
|  |  |  | |  |  |  |  |  |  |  |  |
|  |  | |  |  |  |  |  |  |  |  |  |
|  |  | |  |  |  |  |  |  |  |  |  |
|  |  |  | |  |  |  |  |  |  |  |  |
| **Table S3\| Probe set two-color co-localization validation results.**   1. Validation score index. **(b)** Validation score results.   **(a)** Validation score index.   \| Subclass number \| Class type \| \| --- \| --- \| \| 1 \| Valid \| \| 1.5 \| Valid after manual recovery: a borderline case with a pattern similar to a valid pattern of the gene in another cell type \| \| 2.1 \| Invalid: qualitative due to foci found in one channel and not the other \| \| 2.2 \| Invalid : quantitative \| \| 2.3 \| Invalid: qualitative; a conservative classification of a borderline case in manual examination identifies clear spots in one channel in the two color assay. \| \| 2.4 \| Not enough data \| \| 2.5 \| Single color image not available – technical reasons \| \| 2.6 \| 2 color image not available but signal is very consistent with the gene’s signal in a valid cell type \| \| 2.7 \| 2 color image not available due to technical reasons \| \| 3 \| No signal in single color image \|   **(b)** Validation score results.   \| **screenID** \| **FinalStatus** \| **HeLa** \| **hFF** \| **hLF** \| \| --- \| --- \| --- \| --- \| --- \| \| ANCR \| 1.0 \| 1.5 \| 1 \| 2.6 \| \| Anril \| 1.0 \| 1.5 \| 1 \| 3 \| \| GAS5 \| 1.0 \| 1 \| 1 \| 1 \| \| KCNQIOT1 \| 1.0 \| 3 \| 1 \| 1 \| \| lincFOXF1 \| 1.0 \| 1 \| 1 \| 1 \| \| lincMKLN1_A1 \| 1.0 \| 1 \| 1 \| 2.7 \| \| lincSFPQ \| 1.0 \| 1 \| 1 \| 2.4 \| \| MALAT1 \| 1.0 \| 1 \| 1 \| 1 \| \| MEG3 \| 1.0 \| 3 \| 2.6 \| 1 \| \| NEAT1 \| 1.0 \| 1 \| 2.6 \| 2.6 \| \| NR_024412 \| 1.0 \| 1.5 \| 1 \| 2.3 \| \| NR_029435 \| 1.0 \| 1 \| 1.5 \| 1.5 \| \| TERC \| 1.0 \| 1.5 \| 1 \| 2.2 \| \| TUG1 \| 1.0 \| 1.5 \| 2.2 \| 2.2 \| \| XIST \| 1.0 \| 3 \| 2.5 \| 1 \| \| XLOC_002094 \| 1.0 \| 3 \| 3 \| 1 \| \| XLOC_002408 \| 1.0 \| 2.3 \| 3 \| 1.5 \| \| XLOC_002746 \| 1.0 \| 1 \| 2.2 \| 1 \| \| XLOC_003526 \| 1.0 \| 2.2 \| 1 \| 1 \| \| XLOC_004456 \| 1.0 \| 2.4 \| 2.4 \| 1 \| \| XLOC_004803 \| 1.0 \| 2.2 \| 1 \| 1 \| \| XLOC_005151 \| 1.0 \| 1 \| 3 \| 2.2 \| \| XLOC_005764 \| 1.0 \| 1 \| 1 \| 1 \| \| XLOC_006922 \| 1.0 \| 1 \| 1.5 \| 1.5 \| \| XLOC_008174 \| 1.0 \| 1.5 \| 1.5 \| 1 \| \| XLOC_009233 \| 1.0 \| 2.2 \| 1.5 \| 1 \| \| XLOC_009702 \| 1.0 \| 2.1 \| 1 \| 1 \| \| XLOC_010017 \| 1.0 \| 1 \| 1 \| 1 \| \| XLOC_010514 \| 1.0 \| 3 \| 1 \| 1 \| \| XLOC_011185 \| 1.0 \| 3 \| 1 \| 1 \| \| XLOC_011226 \| 1.0 \| 1.5 \| 2.6 \| 2.6 \| \| XLOC_011950 \| 1.0 \| 1 \| 1 \| 3 \| \| XLOC_012599 \| 1.0 \| 1 \| 1 \| 2.6 \| \| XLOC_L2_008203 \| 1.0 \| 1 \| 1 \| 1 \| \| h19 \| 2.0 \| 2 \| 3 \| 3 \| \| lincGARS \| 2.0 \| 2.3 \| 2.2 \| 2.4 \| \| MIAT \| 2.0 \| 2.2 \| 2.2 \| 2.4 \| \| NRON \| 2.0 \| 2 \| 3 \| 3 \| \| XLOC_000304 \| 2.0 \| 2.3 \| 2.5 \| 2.3 \| \| XLOC_002263 \| 2.0 \| 2.1 \| 3 \| 3 \| \| XLOC_004122 \| 2.0 \| 2.3 \| 2.3 \| 2.3 \| \| XLOC_004198 \| 2.0 \| 2.3 \| 2.2 \| 2.3 \| \| XLOC_006198 \| 2.0 \| 2.7 \| 2.7 \| 2.7 \| \| XLOC_008583 \| 2.0 \| 2.2 \| 2.2 \| 2.2 \| \| XLOC_009474 \| 2.0 \| 2.1 \| 3 \| 3 \| \| XLOC_009662 \| 2.0 \| 2.3 \| 2.2 \| 2.5 \| \| XLOC_010202 \| 2.0 \| 2.1 \| 3 \| 3 \| \| XLOC_010556 \| 2.0 \| 2.1 \| 3 \| 3 \| \| XLOC_011264 \| 2.0 \| 2.1 \| 2.7 \| 2.7 \| \| XLOC_012187 \| 2.0 \| 3 \| 3 \| 2.1 \| \| XLOC_012192 \| 2.0 \| 2.1 \| 3 \| 3 \| \| XLOC_013841 \| 2.0 \| 2.1 \| 3 \| 3 \| \| XLOC_009447 \| 3.0 \| 3 \| 3 \| 3 \| \| XLOC_010263 \| 3.0 \| 3 \| 3 \| 3 \| \| XLOC_010853 \| 3.0 \| 3 \| 3 \| 3 \| \| XLOC_012197 \| 3.0 \| 3 \| 3 \| 3 \| \| XLOC_012564 \| 3.0 \| 3 \| 3 \| 3 \| \| XLOC_012980 \| 3.0 \| 3 \| 3 \| 3 \| \| XLOC_014160 \| 3.0 \| 3 \| 3 \| 3 \| \| XLOC_l2_010926 \| 3.0 \| 3 \| 3 \| 3 \| \| XLOC_010709 \| 2.0 \| 2.7 \| 2.7 \| 2.7 \| |  |  | |  |  |  |  |  |  |  |  |

**Table S4| Mitotic cells analysis.**

| **lincRNA name** | **Localization pattern** | **Cell type** | **# of mitotic cells** | **# of mitotic cells with foci on chromosomes** |
| --- | --- | --- | --- | --- |
| XLOC_005764 | II | HeLa | 7 | 0 |
| XLOC_005151 | I | HeLa | 12 | 0 |
| XLOC_006922 | I | HeLa | 6 | 0 |
| XIST | I | hLF | 18 | 0 |
| ANRIL | I | HeLa | 15 | 5 |
| MEG3 | II | HeLa | 10 | 0 |
| XLOC_003526 | III | hLF | 12 | 0 |

**Table S5| Correlation analysis of lncRNAs with CCNA2.** Presented are lncRNA that had a significant score in at least one of the test described in 3.4 Methods. Pearson r specifies the correlation between the lncRNA and CCNA2 that are imaged simultaneously. The mean number of spots per cell is specified for the lncRNA and CCNA2. Kolmogorov-Smirnov (KS) test p-value is calculated between the distributions of lncRNA counts after splitting G1 and G2/S/M by a threshold on CCNA2 counts. (#) refers to the numbers of lncRNA cells in each sub population (G1 or G2/S/M). Regression P-value is the significance of CCNA2 as a predictor in a linear regression to predict the lncRNA level using CCNA2 and cell area as predictors. Cases that were finally considered as significant are marked red.

| **Name** | **cell** | **#Cellls** | **Pearson r** | **lncRNA spots** | **CCNA2**  **spots** | **KS P_val** | **# G1** | **#**  **G2/S/M** | **Regression**  **Pval** |
| --- | --- | --- | --- | --- | --- | --- | --- | --- | --- |
| lincSFPQ | Hela | 25 | 0.68 | 33.10 | 147.00 | NA | 0 | 25 | 2.31E-03 |
| XLOC_011226 | Hela | 137 | 0.66 | 25.90 | 105.00 | 1.73E-03 | 13 | 124 | 1.00E-10 |
| TUG1 | Hela | 30 | 0.58 | 29.70 | 82.90 | 4.18E-01 | 8 | 22 |  |
| XLOC_003526 | hLF | 24 | 0.55 | 6.88 | 6.50 | 1.36E-01 | 23 | 1 |  |
| XLOC_006922 | hLF | 11 | 0.53 | 10.00 | 8.91 | 8.70E-01 | 10 | 1 |  |
| GAS5 | Hela | 117 | 0.46 | 118.00 | 79.80 | 4.10E-03 | 20 | 97 | 7.09E-01 |
| XLOC_011950 | Hela | 126 | 0.45 | 23.00 | 80.60 | 4.62E-01 | 35 | 91 |  |
| lincSFPQ | hFF | 7 | 0.40 | 20.10 | 72.30 | 1.58E-01 | 2 | 5 |  |
| NR_024412 | Hela | 122 | 0.37 | 12.20 | 66.50 | 1.40E-01 | 54 | 68 |  |
| MEG3 | hLF | 20 | 0.35 | 136.00 | 5.75 | NA | 20 | 0 |  |
| NR_029435 | Imr90 | 23 | -0.36 | 9.35 | 15.00 | 9.40E-02 | 18 | 5 |  |
| XLOC_011185 | hFF | 20 | -0.44 | 5.20 | 16.40 | 6.30E-02 | 14 | 6 | 5.02E-02 |

**References**

1. Cabili MN, Trapnell C, Goff L, Koziol M, Tazon-Vega B, Regev A, Rinn JL: **Integrative annotation of human large intergenic noncoding RNAs reveals global properties and specific subclasses.** *Genes & development* 2011, **25:**1915-1927.

2. Zhu J, Sanborn JZ, Diekhans M, Lowe CB, Pringle TH, Haussler D: **Comparative Genomics Search for Losses of Long-Established Genes on the Human Lineage.** *PLoS Comput Biol* 2007, **3:**e247.

3. Guttman M, Amit I, Garber M, French C, Lin MF, Feldser D, Huarte M, Zuk O, Carey BW, Cassady JP, et al: **Chromatin signature reveals over a thousand highly conserved large non-coding RNAs in mammals.** *Nature* 2009, **458:**223-227.

4. Pruitt K, Tatusova T, Maglott D: **Chapter 18, The Reference Sequence (RefSeq) Project. .** In *The NCBI handbook [Internet]*. Bethesda (MD): National Library of Medicine (US), National Center for Biotechnology Information; 2002.

5. Altschul SF, Gish W, Miller W, Myers EW, Lipman DJ: **Basic local alignment search tool.** *Journal of Molecular Biology* 1990, **215:**403-410.

6. Trapnell C, Hendrickson DG, Sauvageau M, Goff L, Rinn JL, Pachter L: **Differential analysis of gene regulation at transcript resolution with RNA-seq.** *Nature Biotechnology* 2013, **31:**46-53.

7. Kelley D, Rinn J: **Transposable elements reveal a stem cell-specific class of long noncoding RNAs.** *Genome Biology* 2012, **13:**R107.

8. Trapnell C, Pachter L, Salzberg SL: **TopHat: discovering splice junctions with RNA-Seq.** *Bioinformatics* 2009, **25:**1105-1111.

9. Hsu F, Kent WJ, Clawson H, Kuhn RM, Diekhans M, Haussler D: **The UCSC Known Genes.** *Bioinformatics* 2006, **22:**1036-1046.

10. Katz YW, Eric T.; Silterra, Jacob; Schwartz, Schraga; Wong, Bang; Mesirov, Jill P.; Airoldi, Edoardo M.; Burge, Christopher B.: **Sashimi plots: Quantitative visualization of RNA sequencing read alignments.** pp. 2013arXiv1306.3466K. eprint arXiv:1306.3466: ARXIV; 2013:2013arXiv1306.3466K.

11. Trapnell C, Williams BA, Pertea G, Mortazavi A, Kwan G, van Baren MJ, Salzberg SL, Wold BJ, Pachter L: **Transcript assembly and quantification by RNA-Seq reveals unannotated transcripts and isoform switching during cell differentiation.** *Nat Biotech* 2010, **28:**511-515.
